# Primary prevention in liver cirrhosis patients with esophageal varicesa: a systematic review and network meta-analysis

**DOI:** 10.3389/fgstr.2026.1754027

**Published:** 2026-04-16

**Authors:** Feng Li, Xiaoyi- Zhang, Jia-li Chi, Xuemei Zhang, Yu-jing Zhou, Han-fang Xu, Kai Zhang, Yi-jing Ren, Xiu-yan Wei

**Affiliations:** Department of Clinical Pharmacy, Shenyang Pharmaceutical University, Shenyang, Liaoning, China

**Keywords:** esophageal variceal bleeding, liver cirrhosis, network meta-analysis, primary prevention, systematic review

## Abstract

**Objective:**

To compare the efficacy and safety of different primary preventive measures for esophageal variceal bleeding using a network meta-analysis.

**Methods:**

Randomized controlled trials (RCTs) on primary prevention were retrieved from PubMed, Cochrane Library, Embase, and Web of Science. Primary outcomes were the incidence of variceal bleeding, all-cause mortality, All-Cause Bleeding and Bleeding-Related Mortality. Secondary outcomes included adverse events and other decompensation events. Study quality was assessed with the Cochrane Risk of Bias tool. Data were analyzed using Revman 5.4 and Stata 16.0.

**Results:**

A total of 50 RCTs (6510 patients) evaluating 16 interventions were included. For reducing bleeding incidence, the combinations Midodrine + Propranolol (Mido+PPL), Carvedilol + Endoscopic Variceal Band Ligation (Carv+EVBL), and Fuzheng Huayu Capsule + Propranolol (FZHYJN+PPL) were superior. For lower all-cause mortality, Carv+EVBL, Mido+PPL, and Nadolol + Isosorbide Mononitrate (Nado+ISMN) were favorable. Carv+EVBL, EVBL, and Endoscopic Injection Sclerotherapy (EIS) were better for reducing bleeding-related mortality. Regarding safety, Mido+PPL, Nadolol (Nado), and Carvedilol (Carv) had fewer general adverse events, while Isosorbide Mononitrate (ISMN), Mido+PPL, and Carv had lower rates of other decompensation events.

**Conclusion:**

Based on low-certainty evidence, Mido+PPL demonstrated a favorable balance of efficacy and safety and is recommended as a preferred option. Mido+PPL, Carv+EVBL, and FZHYJN+PPL are recommended for primary prophylaxis in cirrhosis. For Child-Pugh A/B patients, Mido+PPL, Carv+EVBL, and FZHYJN+PPL are suggested, with EVBL as an alternative for those intolerant to drugs. EVBL may be considered for Child-Pugh C patients. Mido+PPL is recommended for prophylaxis within one year, FZHYJN+PPL for up to two years, and Carvedilol (alone or with EVBL) for long-term management beyond two years. Carvedilol and Propranolol showed superior net clinical benefit over Nadolol and Timolol. Although Nadolol (alone or with ISMN) may reduce mortality, its higher adverse event rate precludes it as a first-line strategy. FZHYJN, while effective, may increase the risk of other decompensation events.

## 1 Introduction

Cirrhosis represents an advanced, relentlessly progressive hepatic disorder, fundamentally characterized by the extinction of viable hepatocytes after recurrent injury inflicted by persistent insults—ranging from viral, toxic-alcoholic to metabolic triggers—and their replacement by fibrotic parenchyma and regenerative nodule (1). Decompensated cirrhosis is clinically heralded by the accumulation of fluid within the peritoneal cavity and a heightened tendency toward bleeding episodes (2). In clinical practice, liver cirrhosis is stratified into two sequential stages: the compensated phase (corresponding to Child–Pugh grade A) and the decompensated phase (Child–Pugh grades B or C) (3). The initial episode of esophageal variceal bleeding in patients with liver cirrhosis is associated with a one-year rebleeding rate of up to 70% and a mortality rate of up to 33% (4). Esophageal variceal bleeding, one of the most lethal complications of cirrhosis, evolves under sustained portal hypertension: collateral veins become progressively tortuous, dilated and thin-walled until transmural tension exceeds its rupture threshold or is precipitated by external triggers such as coarse food or forceful vomiting (4). Current clinical practice advocates a “detect early, prevent early” paradigm, delivering primary prophylaxis before esophageal varices rupture or bleed, thereby halting progression to overt decompensation (5). A wide range of drugs are now prescribed for primary prophylaxis against variceal bleeding in cirrhosis; although some have proven efficacy, robust evidence to guide their routine clinical use remains incomplete (6).《Expert consensus on the diagnosis and treatment of esophageal and gastric variceal rupture bleeding in cirrhotic portal hypertension (2024 edition)》recommends a risk-stratified pharmacologic strategy: statins for patients without clinically significant portal hypertension, non-selective beta-blockers (NSBB) (e.g., Propranolol) for mild esophageal varices, and carvedilol for those with raised hepatic venous pressure gradient or advanced disease (7).《AASLD Practice Guidance: Risk Stratification and Management of Portal Hypertension and Varices in Cirrhosis》recommends NSBB for patients with small esophageal varices, whereas either NSBB or EVBL may be chosen for individuals with large varices (8). 《Guidelines on the management of esophagogastric variceal bleeding in cirrhotic portal hypertension(2023)》 states that no established therapy exists for mild esophageal varices, while NSBB are recommended for patients with larger mild varices (9). AASLD guidance and the Baveno VI consensus state that either NSBB or EVBL may be used for primary prophylaxis in patients with high-risk esophageal varices (large or small varices displaying red-color signs) (10, 11).《UK guidelines on the management of variceal hemorrhage in cirrhotic patients(2015)》positions NSBB—namely nadolol and carvedilol—and EVBL as the two preferred first-line options for primary prophylaxis of variceal bleeding (12).

Current international guidelines do not provide tailored recommendations for each variceal grade or cirrhosis stage, offer no comparative ranking of combination strategies, and establish no explicit hierarchy among the individual NSBB (13). Although an increasing number of randomized trials have recently refined primary prophylaxis of esophageal variceal bleeding, an updated network meta-analysis directly comparing combination pharmacological strategies is still lacking (14). We therefore conducted a systematic review with network meta-analysis to compare the efficacy and safety of combination pharmacological regimens for primary prevention of esophageal varices across different stages of cirrhosis, and to establish a hierarchical ranking of these strategies to inform the optimal treatment choice for each disease phase.

## 2 Materials and methods

### 2.1 Data sources

Data were systematically retrieved from PubMed, the Cochrane Library, Embase, and Web of Science databases to identify RCTs evaluating primary prophylaxis strategies for esophageal variceal bleeding in patients with liver cirrhosis. The search spanned from the inception of each database to September 2025. A comprehensive search strategy was employed, integrating both MeSH terms and free-text keywords. The primary English search terms included “Liver Cirrhosis, “ “esophageal varices, “ and related variants.

### 2.2 Inclusion and exclusion criteria

Inclusion criteria:

1. Participants: Adult patients diagnosed with liver cirrhosis and esophageal varices, or at high risk of variceal bleeding, with no prior history of variceal hemorrhage.
2. Interventions: NSBB including propranolol, carvedilol, nadolol, and timolol; isosorbide mononitrate(ISDM); EVBL; endoscopic injection sclerotherapy(EIS); Fuzheng Huayu capsules(FZHYJN); simvastatin(Simv); Propranolol+ Endoscopic Variceal Band Ligation(PPL+EVBL); Carvedilol**+**Endoscopic Variceal Band Ligation(Carv+EVBL); Nadolol**+**Endoscopic Variceal Band Ligation(Nado+EVBL); Nadolol+Isosorbide Mononitrate(Nado+ISMN); Fuzheng Huayu Capsule+ Propranolol(FZHYJN+PPL); Midodrine+ Propranolol(Mido+PPL).
3. Comparisons: Placebo control or head-to-head comparisons between different interventions.
4. Outcomes:
  - Efficacy outcomes: All-cause bleeding rate, esophageal variceal bleeding rate, overall mortality, and bleeding-related mortality.
  - Safety outcomes: Incidence of general adverse events, occurrence of other decompensation events, and liver transplantation rate.
  - Exploratory outcome: Comparative efficacy ranking of FZHYJN.
5. Study design: RCTs, published in either English or Chinese.

Exclusion Criteria:

1. Individuals with a documented history of variceal hemorrhage, either current or previous.
2. Participants who had previously undergone liver transplantation.
3. Trials evaluating secondary prophylaxis or recruiting subjects already in receipt of primary prophylaxis for esophageal varices.
4. Publications in which primary endpoint data were absent or could not be extracted.
5. Studies characterized by duplicate publication, questionable data integrity, or insufficient methodological detail.
6. Investigations with incompletely reported outcome measures or for which full-text manuscripts were unobtainable.
7. Non-randomized designs, quasi-experimental studies, and purely observational investigations.

### 2.3 Study registration

This review was conducted in accordance with the Preferred Reporting Items for Systematic Reviews and Meta-Analyses (PRISMA) statement and was prospectively registered with the International Prospective Register of Systematic Reviews (PROSPERO; registration identifier CRD42024496835).

### 2.4 Data extraction and quality appraisal

Study design, participant-level variables, and preventive interventions were independently abstracted by two reviewers onto a piloted, standardized form. Methodological quality was appraised in duplicate with the Cochrane risk-of-bias tool, examining: (1) random-sequence generation, (2) allocation concealment, (3) blinding of participants and personnel, (4) blinding of outcome assessors, (5) completeness of outcome data, (6) selective outcome reporting, and (7) any additional domains of concern. After parallel assessment, results were cross-checked; discrepancies were resolved by consensus or arbitration by a third senior investigator.

### 2.5 Statistical analysis

All analyses and graphics were generated with Stata 16 and Review Manager 5.3. The Bayesian network meta-analysis was employed to pool direct and indirect evidence; the random-effects models were used throughout the entire process. Continuous outcomes were summarized as standardized mean differences (SMDs), whereas dichotomous endpoints were expressed as odds ratios (ORs) with 95% credible intervals (CrIs). Interventions were hierarchically ranked using the surface under the cumulative ranking curve (SUCRA). Heterogeneity was quantified with *I²* statistics and corresponding *P* values. Publication bias was explored qualitatively via funnel-plot asymmetry and Egger’s regression test.

## 3 Results

### 3.1 Literature search results

**Figure 1:**
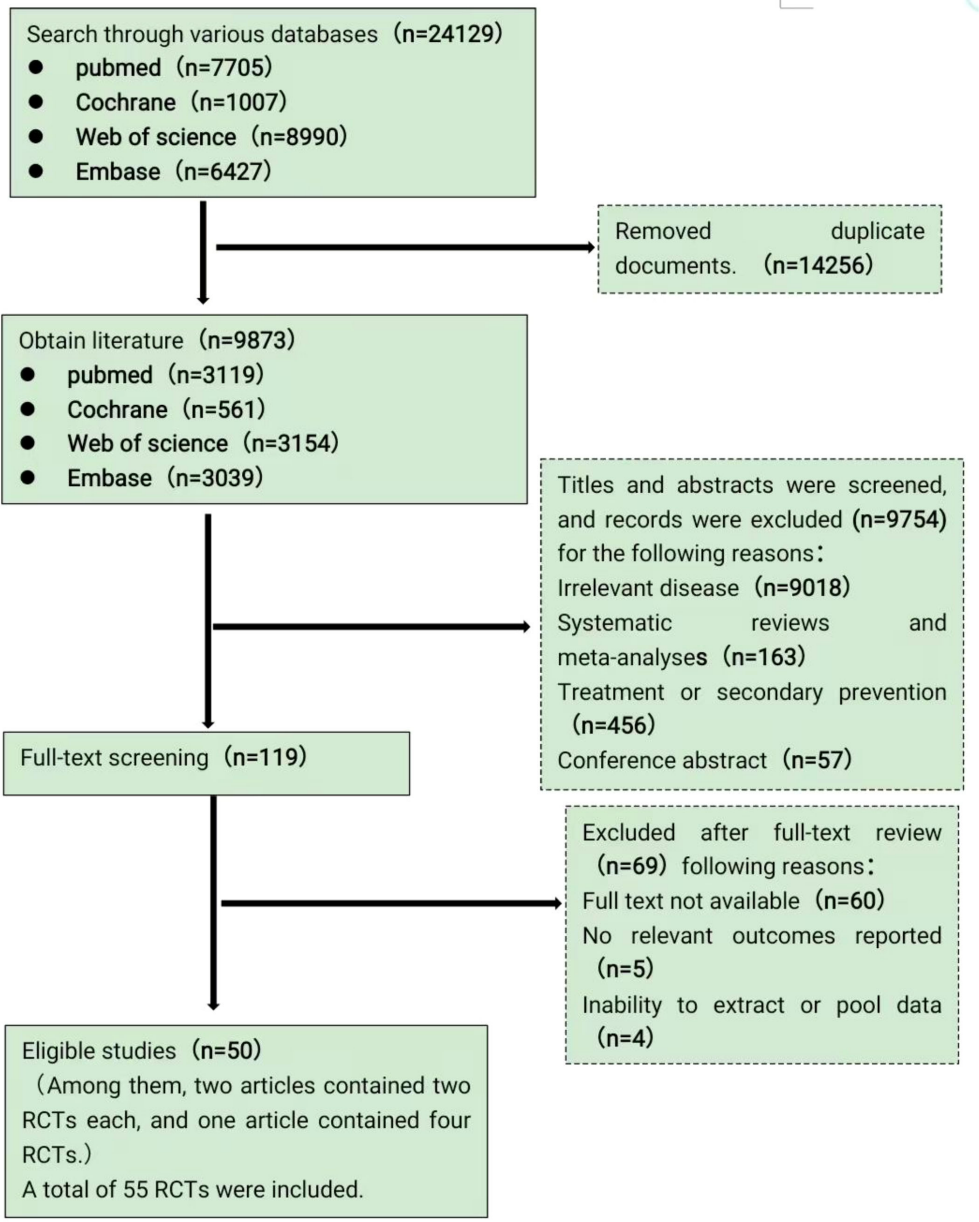
Literature screening flow chart.

Database queries retrieved a total of 24129 citations, after deduplication 9873 unique records remained. Title-and-abstract screening identified 119 articles for full-text appraisal, of which 50 publications fulfilled eligibility criteria. These reports contained 55 discrete randomized controlled trials (two articles contributed two RCTs and one article contributed four RCTs). The PRISMA flow diagram details the complete selection process. The PRISMA flow diagram details the complete selection process **Figure 1**.

### 3.2 Characteristics of included trials and participants

The qualitative synthesis comprised 55 RCTs reported in 49 English-language and 1 Chinese-language publications that satisfied all eligibility criteria (13, 15–63). 41 trials had two parallel arms and 8 trials were three-arm studies, yielding a total of 16 distinct prophylactic strategies. Design-level details were summarized in **Table 1**.

**Table 1:** Details of included studies.

Study	Country	Study design	Study population	Intervention	Control group 1	Control group 2	Follow-up time (months)
Abhijeet et al, 2024 (64)	India	RCT	Severe or Mild Varices in Cirrhosis	Mido+PPL	PPL		12
Antonio et al, 1989 (65)	Italy	RCT	NA	EIS	PBO		16.8
Ayman et al, 2018 (66)	Egypt	RCT	Severe Varices in Cirrhosis	EVBL	Carv	PPL	12
Binay et al, 1999 (67)	India	RDBCT	Moderate or Severe Varices in Cirrhosis	EVBL	PPL		17.6
C Merkel et al, 1996 (44)	Italy	MRSBCT	Mild, Moderate, or Severe Varices in Cirrhosis	Nado	Nado+ISDM		40
C Triantos et al, 2005 (60)	Greece/UK	multi-center RCT	Severe or Mild Varices in Cirrhosis	EVBL	PBO		24
Carlo et al, 2000 (68)	Italy	Single-blind multi-center RCT	Mild, Moderate, or Severe Varices in Cirrhosis	Nado	Nado+ISDM		30
Chii et al, 1997 (69)	China	RCT	NA	EVBL	PBO		24
Chii et al, 2016 (70)	China	RCT	NA	EVBL	PPL		36
Danielle et al, 2015 (71)	Brazil	Single-blind RCT	Moderate or Severe Varices in Cirrhosis	EVBL+PPL	EVBL		12
David E et al, 2021 (72)	America	Multi-center Double-blind RCT	NA	Simv	PBO		24
Dhiraj et al, 2009 (73)	Scotland	multi-center RCT	Severe Varices in Cirrhosis	EVBL	Carv		24
Didier et al, 1988 (74)	France	RCT	NA	Nado	PBO		12
Dimitrios et al, 2005 (75)	Greece	RCT	Moderate or Severe Varices in Cirrhosis	EVL	PPL		27.5
Gaetano et al, 1988 (76)	Italy	Single-blind RCT	NA	Nado	PBO		24.4
Gianmario et al, 2002 (77)	Italy	RCT	Severe Varices in Cirrhosis	ISDM	Nado		24
Gin et al, 1999 (78)	China	RCT	Moderate or Severe Varices in Cirrhosis	EVBL	EIS		29
Gin et al, 2004 (79)	China	RCT	Moderate or Severe Varices in Cirrhosis	EVBL	Nado		
Gin et al, 2010 (80)	China	RCT	Mild, Moderate, or Severe Varices in Cirrhosis	EVBL+Nado	Nado		26
Guido et al, 1988 (81)	Italy	RCT	NA	EIS	PBO		13
Haroldo et al, 1990 (82)	Spain	RCT	Mild, Moderate, or Severe Varices in Cirrhosis	PPL	PBO		12
Harsh et al, 2024 (83)	India	RCT	Moderate or Severe Varices in Cirrhosis	Caev+EVBL	EVBL	Carv	12
Hasnain et al, 2014 (84)	Pakistan	RCT	Moderate or Severe Varices in Cirrhosis	EVBL	Carv		24
Henk et al, 2003 (85)	the Netherlands	multi-center RCT	Mild, Moderate, or Severe Varices in Cirrhosis	EIS	PBO		32
Hockf et al, 2002 (86)	Italy	multi-center RCT	Moderate or Severe Varices in Cirrhosis	EVBL	PPL	ISDM	20
Huay et al, 2006 (87)	China	RCT	Moderate or Severe Varices in Cirrhosis	EVBL	Nado+ISDM		23
Jean et al, 1987 (35)	France	RCT	Moderate or Severe Varices in Cirrhosis	PPL	PBO		24
Jie(1)et al, 2013 (88)	China	RDBCT	Mild Varices in Cirrhosis	FZHYJN	PBO		24
Jie(2) et al, 2013 (88)	China	RDBCT	Moderate or Severe Varices in Cirrhosis	FZHYJN	PPL	FZHYJN+PPL	24
K.J et al, 1982 (89)	France	RCT	NA	EIS	PBO		6
Lin et al, 2025 (90)	China	RCT	NA	EVBL	PPL		12
Lorenzo et al, 2007 (91)	Italy	RCT	Moderate or Severe Varices in Cirrhosis	EVBL	PPL		40
Lothar et al, 1985 (92)	Germany	RCT	Mild, Moderate, or Severe Varices in Cirrhosis	EIS	PBO		25
Mario et al, 1997 (93)	Italy	RCT	NA	ISDM	PPL		24
Michael et al, 2004 (94)	Germany	RCT	Moderate or Severe Varices in Cirrhosis	EVBL	PPL		24
Morimasa et al, 2004 (95)	Japan	RCT	NA	EIS	PPL		13
Muhammad et al, 2023 (96)	Pakistan	RCT	Moderate or Severe Varices in Cirrhosis	Carv	PPL		36
P. Svoboda et al, 1999 (47)	Czechia	RCT	Mild, Moderate, or Severe Varices in Cirrhosis	EIS	EVBL	PBO	24
Paul et al, 2005 (97)	America	RCT	NA	EVBL	PPL		36
Pavel et al, 2011 (98)	Czechia	RCT	Severe Varices in Cirrhosis	EVBL	PPL		18
Pérez et al, 2010(49)	Chile	RCT	NA	EVBL	PPL		24
Roberto et al, 2005 (99)	Europe	multi-center RCT	NA	Timo	PBO		72
Rome et al, 2005 (100)	America	multi-center RCT	Mild, Moderate, or Severe Varices in Cirrhosis	EVBL	PPL		24
Shiv et al, 1999 (101)	India	RCT	Mild, Moderate, or Severe Varices in Cirrhosis	EVBL	PPL		18
Thierry(1)et al, 1991 (102)	France	RCT	Moderate or Severe Varices in Cirrhosis	PPL	PBO		24
Thierry(2)et al, 1991 (102)	France	RCT	Severe Varices in Cirrhosis	PPL	PBO		24
Thierry(3)et al, 1991 (102)	France	RCT	Severe Varices in Cirrhosis	Nsdo	PBO		24
Thierry(4)et al, 1991 (102)	France	RCT	Moderate or Severe Varices in Cirrhosis	Nsdo	PBO		24
Thomas et al, 2013 (103)	Austria	RCT	Mild or Moderate Varices in Cirrhosis	PPL	Carv	EVBL	24
Toni et al, 1990 (104)	France	RCT	Mild, Moderate, or Severe Varices in Cirrhosis	EIS	PPL	PBO	24
Tsung et al, 2024 (105)	China	RDBCT	Moderate or Severe Varices in Cirrhosis	EVBL	PPL		36
Virendra et al, 2022 (106)	India	RCT	NA	EVBL	PPL		12
William et al, 1988 (107)	America	RCT	NA	EIS	PBO		95
Xiao(1)et al, 2014 (15)	China	RCT	Mild Varices in Cirrhosis	FZHYJN	PBO		24
Xiao(2)et al, 2014 (15)	China	RCT	Severe Varices in Cirrhosis	FZHYJN	FZHYJN+PPL	PPL	24

Across the 55 trials, 6510 participants were randomized (male, n=4140; female, n=1864). Mean age ranged from 41 to 62 years; 4 studies did not provide sex-stratified data. Etiologies of cirrhosis were predominantly alcohol-related or viral. Child–Pugh classification was extractable for 40 trials: class A (n = 1847), class B (n = 1684) and class C (n =1080); the remaining 15 trials did not report functional liver-status scores. Patient-level characteristics were presented in **Table 2**.

**Table 2:** Basic characteristics of included patients.

Study	Mean age	Male ratio(%)	Etiology (alcohol/viral/other)	Child-paugh (A/B/C) number	Total number
Abhijeet et al, 2024 (64)	52.2	79.2	54/16/70	0/61/79	140
Antonio et al, 1989 (65)	61.8	58	0/0/31	20/15/6	41
Ayman et al, 2018 (66)	51.2	69.7	0/258/0	68/82/150	264
Binay et al, 1999 (67)	40.4	73.3	5/25/0	11/15/4	30
C Merkel et al, 1996 (44)	57.5	62.3	79/53/14	NA	146
C Triantos et al, 2005 (60)	61.5	73.1	18/18/16	17/13/22	52
Carloet al, 2000 (68)	57.5	62.3	79/53/14	NA	146
Chii et al, 1997 (69)	55.5	80.1	23/96/7	33/45/48	126
Chii et al, 2016 (70)	55.5	78	21/73/6	45/39/16	100
Danielle et al, 2015 (71)	54.5	71.2	17/35/14	41/17/8	66
David E et al, 2021 (72)	NA	NA	NA	0/0/149	149
Dhiraj et al, 2009 (73)	54.3	65.1	99/0/53	73/49/78	152
Didier et al, 1988 (74)	56	74.5	78/12/16	62/44/0	106
Dimitrios et al, 2005 (75)	60.4	70	15/33/12	28/24/8	60
Gaetano et al, 1988 (76)	53.4	73.7	30/0/0	0/43/14	57
Gianmario et al, 2002 (77)	60	71.2	13/27/12	NA	52
Gin et al, 1999 (78)	56	84.3	38/82/7	36/55/36	127
Gin et al, 2004 (79)	56	77	20/73/7	46/38/16	100
Gin et al, 2010 (80)	55.8	62.1	24/101/15	75/42/23	140
Guido et al, 1988 (81)	56.5	70.7	47/100/19	32/69/39	140
Haroldo et al, 1990 (82)	54	71.6	80/NA	59/35/8	102
Harsh et al, 2024 (83)	51.4	85.1	93/35/48	NA	330
Hasnain et al, 2014 (84)	47.8	72.6	3/151/14	74/72/22	168
Henk et al, 2003 (85)	55.5	57.2	81/45/74	91/57/18	166
Hockf et al, 2002 (86)	55.4	56.4	112/0/70	50/66/56	172
Huay et al, 2006 (87)	61	62.3	11/47/3	27/26/8	61
Jean et al, 1987 (35)	54.1	NA	207/NA/MA	NA	230
Jie(1) et al, 2013 (88)	49.5	83.9	NA	41/13/2	56
Jie(2) et al, 2013 (88)	50	73.3	NA	35/22/3	90
K.J et al, 1982 (89)	NA	NA	NA	NA	65
Lin et al, 2025 (90)	55.3	70.6	NA	73/42/11	126
Lorenzo et al, 2007 (91)	52.5	NA	0/49/44	NA	62
Lothar et al, 1985 (92)	52.6	66	88/16/5	31/48/21	109
Mario et al, 1997 (93)	58	60.2	21/NA/NA	NA	118
Michael et al, 2004 (94)	55.8	68.4	78/47/27	71/62/19	152
Morimasa et al, 2004 (95)	58.9	60	2/23/0	7/18/0	25
Muhammad et al, 2023 (96)	54	53.3	200/12/0	79/99/34	220
P. Svoboda et al, 1999 (47)	46.3	77.7	109/48/0	92/46/19	157
Paul et al, 2005 (97)	51.8	54.5	6/13/12	NA	31
Pavel et al, 2011 (98)	56.5	71	46/9/8	38/30/5	73
Pérez et al, 2010 (49)	59	49	18/9/48	40/29/6	75
Roberto et al, 2005 (99)	45	59	51/142/20	189/24	213
Rome et al, 2005 (100)	54.6	29	7/47/16	NA	62
Shiv et al, 1999 (101)	41.5	73	20/38/31	16/45/28	89
Thierry(1)et al, 1991 (102)	54	71	205/13/12	NA	230
Thierry(2)et al, 1991 (102)	54	70	61/24/89	NA	174
Thierry(3)et al, 1991 (102)	54	72	41/12/26	NA	79
Thierry(4)et al, 1991 (102)	56	75	76/12/18	NA	106
Thomas et al, 2013 (103)	53	77	57/34/13	66/30/8	104
Toni et al, 1990 (104)	56.2	60.3	100/NA/NA	29/61/32	126
Tsung et al, 2024 (105)	62.5	75.7	20/125/18	59/67/18	144
Virendra et al, 2022 (106)	49.5	76.9	81/31/48	0/102/58	160
William et al, 1988 (107)	41.5	73.7	65/6/24	NA	95
Xiao(1)et al, 2014 (15)	49.5	83.9	NA	41/13/2	56
Xiao(2)et al, 2014 (15)	50	73.3	NA	52/32/6	90

NA: Not Available.

### 3.3 Risk of bias assessment results

**Figure 2:**
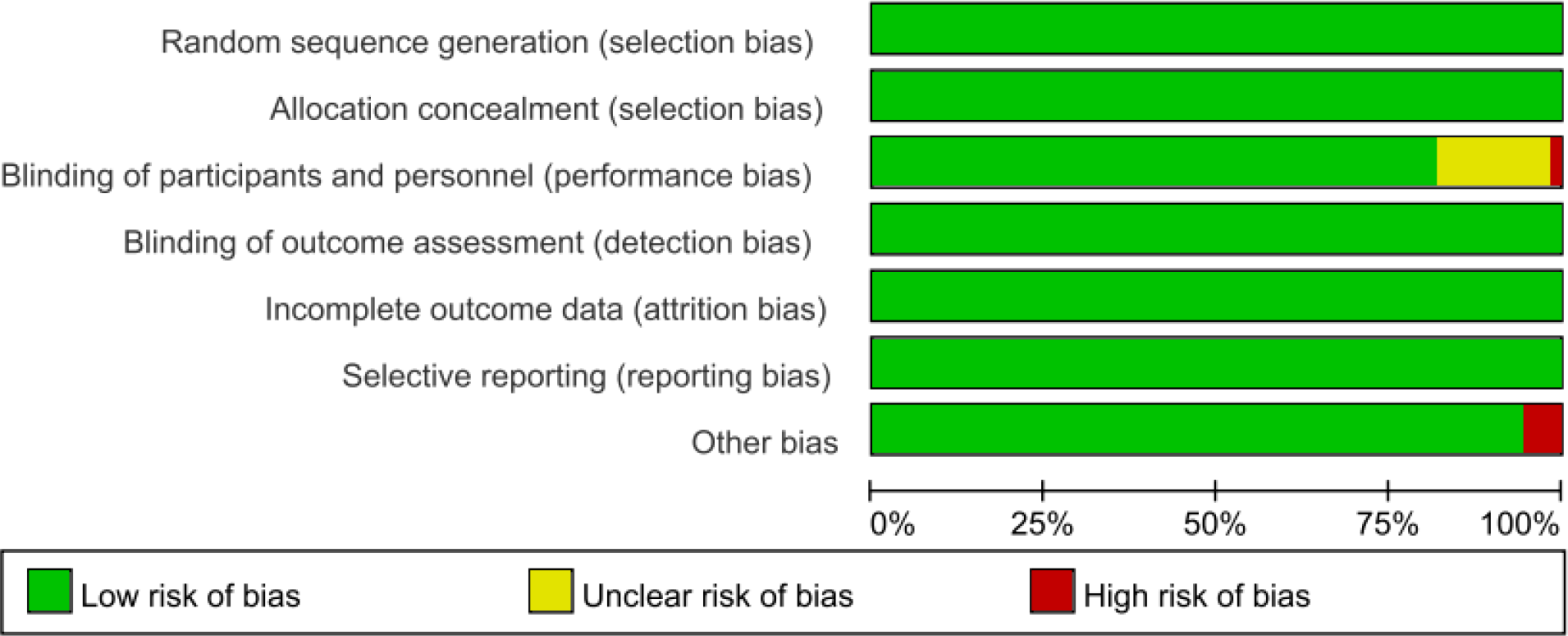
Risk of bias graph.

**Figure 3:**
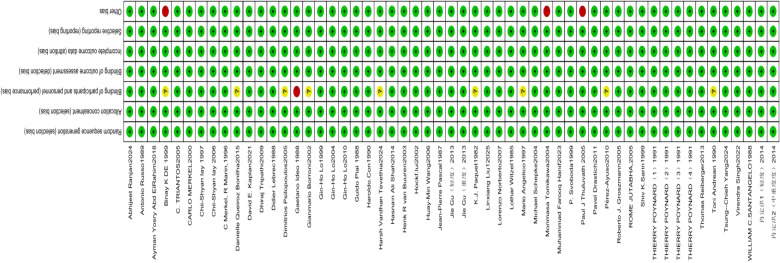
Risk of bias summary graph.

The methodological quality of the 55 trials was summarized graphically in **Figures 2**, **3**. All trials reported an adequate method for generating the random sequence (e.g., random-number tables, computer-generated sequences or sealed envelopes) and were rated at low risk of selection bias. Allocation concealment was explicitly described in every study and likewise judged as low risk. With respect to blinding, 9 trials provided insufficient information and were classified as unclear risk (18, 19, 24, 27, 43, 48, 49, 56, 59),whereas 1 open-label study was deemed high risk (26). Outcome data were complete in all 55 trials, yielding a low risk of attrition bias. No evidence of selective outcome reporting was detected in any trial. Among other potential threats, 3 studies were considered high risk because of small sample sizes (18, 45, 58). Overall, the evidence base was characterized by a low risk of bias and satisfactory methodological rigor.

### 3.4 Network meta-analysis results

#### 3.4.1 All-cause bleeding rate

##### 3.4.1.1 Network geometry

**Figure 4:**
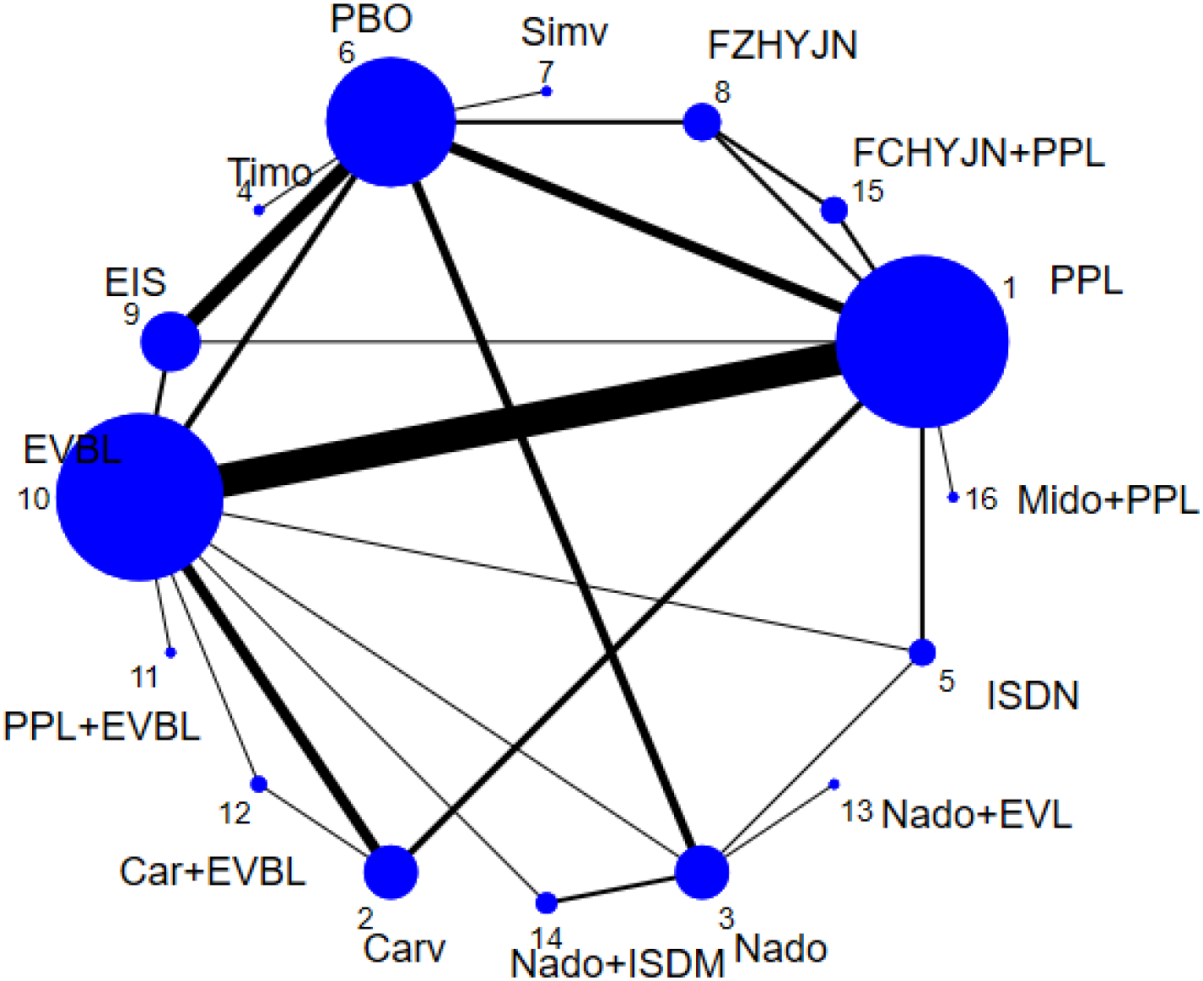
Network graph of all-cause bleeding rates.

All-cause bleeding rate was defined in this study as any clinically confirmed variceal hemorrhage occurring in the esophageal or gastric fundal regions. A total of 54 studies reported outcomes related to all-cause bleeding rates, including 2 single-arm studies, 46 two-arm studies, and 6 three-arm studies. These studies involved 6433 patients and evaluated 16 prophylactic interventions. The network diagram was presented in **Figure 4**.

##### 3.4.1.2.Network meta-analysis results and probability rankings

**Figure 5:**
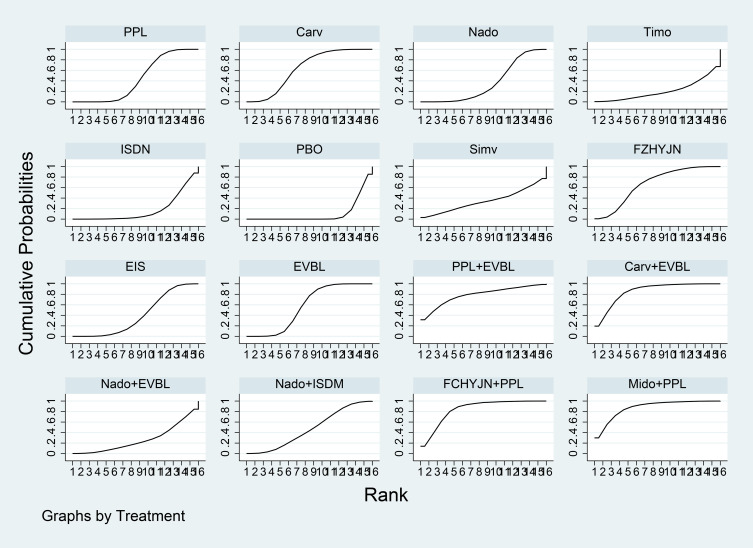
All-Cause bleeding rates SUCRA plot.

**Table 3** summarized the network meta-analysis results. All active interventions significantly reduced all-cause variceal bleeding compared with placebo. Among them, Mido+PPL, Carv+EVBL and FZHYJN+PPL showed the largest reductions, with Mido+PPL exhibiting the lowest event rate for primary prophylaxis. Within the NSBB class, the hierarchy Carv > PPL > Nado > Timo was statistically robust. **Figure 5** displayed the cumulative ranking curves; larger surface under the cumulative ranking curve (SUCRA) indicated greater likelihood of benefit. The SUCRA-based ranking for prevention of all-cause variceal bleeding was: Mido+PPL > Carv+EVBL > FZHYJN+PPL > PPL+EVBL > FZHYJN > Carv > EVBL > Nado+ISMN > PPL > EIS > Nado > Simv > Nado+EVBL >Timo > ISDN > PBO.

**Table 3:** League table for all-cause bleeding rates.

Mido+PPL															
0.84 (0.09, 7.74)	Carv+EVBL														
0.73 (0.08, 6.23)	0.86 (0.12, 6.08)	FZHYJN+PPL													
0.84 (0.03, 20.78)	1.00 (0.05, 20.76)	1.15 (0.06, 23.93)	PPL+EVBL												
0.35 (0.05, 2.54)	0.41 (0.07, 2.42)	0.47 (0.12, 1.83)	0.41 (0.02, 7.62)	FZHYJN											
0.30 (0.05, 1.85)	0.35 (0.09, 1.43)	0.41 (0.09, 1.82)	0.35 (0.02, 5.69)	0.86 (0.25, 3.03)	Carv										
0.25 (0.04, 1.40)	0.29 (0.07, 1.18)	0.34 (0.08, 1.35)	0.29 (0.02, 4.34)	0.71 (0.23, 2.19)	0.83 (0.43, 1.59)	EVBL									
0.20 (0.03, 1.54)	0.23 (0.04, 1.41)	0.27 (0.05, 1.57)	0.24 (0.01, 4.38)	0.57 (0.12, 2.67)	0.66 (0.18, 2.41)	0.81 (0.26, 2.48)	Nado+ISDM								
0.19 (0.04, 1.04)	0.23 (0.05, 0.96)	0.26 (0.07, 1.00)	0.23 (0.01, 3.50)	0.56 (0.19, 1.61)	0.64 (0.32, 1.28)	0.78 (0.51, 1.19)	0.97 (0.30, 3.09)	PPL							
0.17 (0.03, 1.08)	0.20 (0.04, 0.97)	0.23 (0.05, 1.04)	0.20 (0.01, 3.31)	0.49 (0.14, 1.69)	0.57 (0.22, 1.47)	0.69 (0.34, 1.43)	0.86 (0.25, 3.02)	0.89 (0.43, 1.86)	EIS						
0.15 (0.02, 1.02)	0.18 (0.04, 0.92)	0.21 (0.04, 1.00)	0.18 (0.01, 3.06)	0.44 (0.12, 1.65)	0.51 (0.18, 1.44)	0.62 (0.27, 1.42)	0.77 (0.28, 2.10)	0.80 (0.34, 1.88)	0.90 (0.35, 2.33)	Nado					
0.12 (0.00, 3.24)	0.14 (0.01, 3.33)	0.16 (0.01, 3.71)	0.14 (0.00, 7.12)	0.34 (0.02, 6.92)	0.40 (0.02, 7.30)	0.48 (0.03, 8.30)	0.60 (0.03, 12.21)	0.62 (0.04, 10.64)	0.70 (0.04, 12.01)	0.77 (0.04, 14.02)	Simv				
0.10 (0.01, 1.20)	0.12 (0.01, 1.16)	0.14 (0.01, 1.29)	0.12 (0.00, 3.07)	0.29 (0.04, 2.31)	0.34 (0.05, 2.27)	0.41 (0.07, 2.48)	0.51 (0.08, 3.36)	0.53 (0.09, 3.22)	0.59 (0.09, 3.80)	0.66 (0.13, 3.25)	0.85 (0.03, 23.29)	Nado+EVBL			
0.07 (0.00, 1.09)	0.08 (0.01, 1.09)	0.09 (0.01, 1.21)	0.08 (0.00, 2.64)	0.20 (0.02, 2.19)	0.23 (0.02, 2.25)	0.28 (0.03, 2.51)	0.34 (0.03, 3.87)	0.36 (0.04, 3.22)	0.40 (0.04, 3.64)	0.44 (0.05, 4.31)	0.57 (0.02, 19.16)	0.67 (0.04, 10.84)	Timo		
0.08 (0.01, 0.58)	0.10 (0.02, 0.55)	0.11 (0.02, 0.59)	0.10 (0.01, 1.75)	0.24 (0.06, 1.01)	0.28 (0.08, 0.90)	0.33 (0.12, 0.93)	0.42 (0.10, 1.75)	0.43 (0.16, 1.16)	0.48 (0.15, 1.59)	0.54 (0.17, 1.74)	0.69 (0.03, 13.82)	0.81 (0.11, 5.91)	1.21 (0.11, 13.20)	ISDN	
0.07 (0.01, 0.39)	0.08 (0.02, 0.35)	0.09 (0.02, 0.37)	0.08 (0.01, 1.24)	0.19 (0.06, 0.59)	0.22 (0.10, 0.51)	0.27 (0.15, 0.48)	0.33 (0.11, 1.06)	0.35 (0.19, 0.61)	0.39 (0.21, 0.70)	0.43 (0.20, 0.95)	0.56 (0.03, 9.06)	0.65 (0.11, 3.89)	0.97 (0.12, 8.17)	0.80 (0.27, 2.39)	PBO

##### 3.4.1.3 Consistency assessment

Local inconsistency was examined with the node-splitting approach; comparisons yielding *P* < 0.05 were considered discordant and excluded from pooling. As shown in **Table 4**, all node-split analyses returned *P* values > 0.05, indicating satisfactory consistency across direct and indirect evidence loops.

**Table 4:** Local inconsistency test table for all-cause bleeding rates.

Side	Direct		Indirect		Difference		*P* value
	Coef.	Std. err.	Coef.	Std. err.	Coef.	Std. err.	
PPL vs Carv	-0.3187	0.490453	-0.58158	0.525068	0.262881	0.718061	0.714
PPL vs ISDN	0.383781	0.573271	2.210262	0.996789	-1.82648	1.154306	0.114
PPL vs PBO	1.216804	0.418068	0.917636	0.414254	0.299168	0.58437	0.609
PPL vs FZHYJN	-0.43779	0.654498	-0.93337	0.98831	0.49558	1.18543	0.676
PPL vs EIS	1.613751	1.04813	-0.1043	0.401429	1.71805	1.124406	0.127
PPL vs EVBL	-0.26229	0.250707	-0.20291	0.454051	-0.05938	0.518189	0.909
PPL vs FZHYJN+PPL	-1.26159	0.702588	-2.25275	2.301518	0.99116	2.37086	0.676
Carv vs EVBL	0.246236	0.387939	0.014294	0.707773	0.231942	0.80625	0.774
Carv vs Carv+EVBL	-1.33022	0.794113	0.121273	1.544836	-1.45149	1.710006	0.396
Nado vs ISDN	1.911719	1.08336	0.072287	0.702396	1.839432	1.291135	0.154
Nado vs PBO	0.822655	0.51604	0.886013	0.665953	-0.06336	0.83974	0.94
Nado vs EVBL	-0.63252	0.857525	-0.41412	0.493448	-0.2184	0.989363	0.825
Nado vs Nado+EVBL	0.41723	0.814683	-0.46668	1564.531	0.883906	1564.531	1
Nado vs Nado+ISDM	-0.5211	0.596803	0.504851	1.009555	-1.02595	1.172843	0.382
Timo vs PBO	0.028988	1.086485	1.506326	3700.904	-1.47734	3700.905	1
ISDN vs EVBL	-1.3114	0.968751	-0.99604	0.657586	-0.31536	1.200762	0.793
PBO vs Simv	-0.58345	1.421948	-2.22126	4560.695	1.637816	4560.695	1
PBO vs FZHYJN	-1.96603	0.940584	-1.47045	0.721484	-0.49558	1.18543	0.676
PBO vs EIS	-1.0168	0.331481	-0.54507	0.806291	-0.47173	0.871173	0.588
PBO vs EVBL	-0.90061	0.52809	-1.51659	0.368297	0.615984	0.647868	0.342
FZHYJN vs FZHYJN+PPL	-0.8238	0.716335	0.167364	2.288674	-0.99116	2.370861	0.676
EIS vs EVBL	-0.1039	0.588469	-0.53962	0.479256	0.435713	0.758776	0.566
EVBL vs PPL+EVBL	-1.22782	1.375709	0.588605	2668.367	-1.81643	2668.367	0.999
EVBL vs Carv+EVBL	-0.93525	0.798624	-2.38674	1.537845	1.451491	1.710006	0.396
EVBL vs Nado+ISDM	0.817445	0.897831	-0.20849	0.754621	1.02594	1.17284	0.382

#### 3.4.2 Esophageal variceal bleeding rate

##### 3.4.2.1 Network geometry

**Figure 6:**
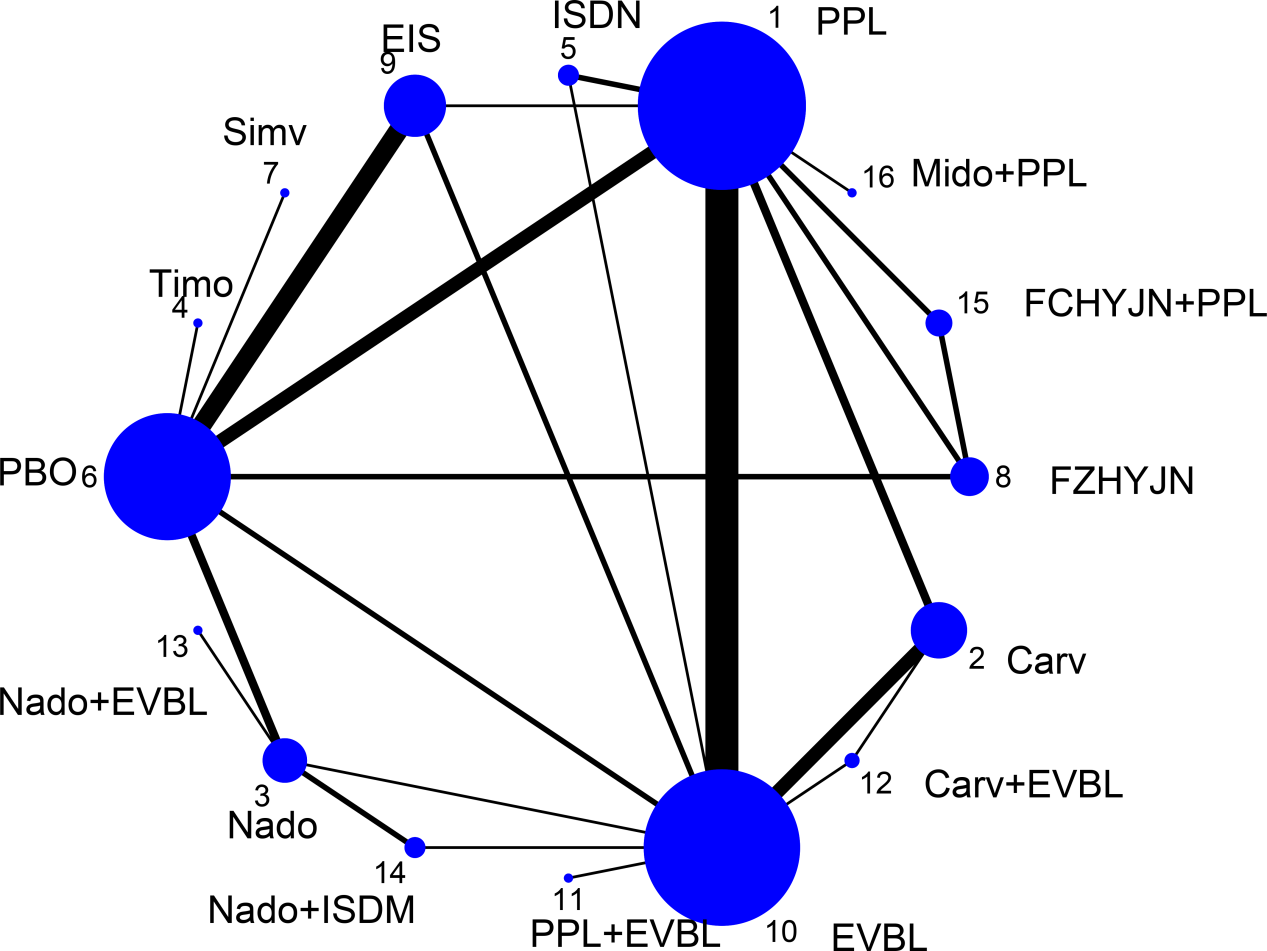
Network graph of EVB rates.

EVB was strictly defined as clinically evident hemorrhage attributable to ruptured esophageal varices; bleeding from gastric or other extra-esophageal sources was excluded. Among the 54 trials that reported all-cause variceal outcomes, 8 trials were removed because of very small sample sizes and evident publication bias (17, 18, 24, 26, 27, 36, 58, 60). The remaining 46 trials (40 two-arm and 6 three-arm) evaluating 16 interventions and enrolling 6100 participants formed the EVB network, the geometry of which was depicted in **Figure 6**.

##### 3.4.2.2 Network meta-analysis results and probability rankings

**Figure 7:**
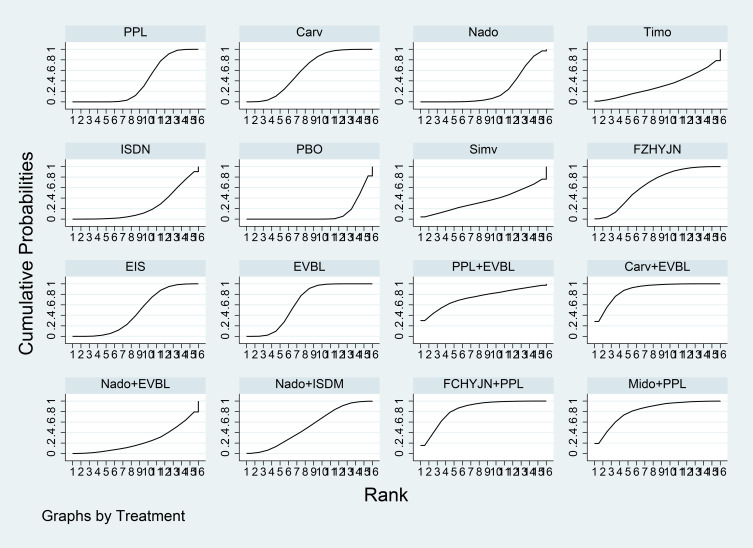
EVB rates SUCRA plot.

Network meta-analysis estimates presented in **Table 5** showed every active intervention to be superior to placebo in reducing EVB, with Carv+EVBL, FZHYJN+PPL and Mido+PPL yielding the largest risk reductions. Among these, Carv+EVBL conferred the lowest EVB incidence for primary prophylaxis. Within the NSBB class the hierarchy Carv > PPL > Timo > Nado was statistically significant. **Figure 7** presented the cumulative ranking curves; larger SUCRA indicated greater expected benefit. The SUCRA-based ranking for EVB prevention was: Carv+EVBL > FZHYJN+PPL > Mido+PPL > PPL+EVBL > FZHYJN > EVBL > Carv > Nado+ISMN > EIS>PPL > Simv >Timo > Nado+EVBL > Nado > ISDN > PBO.

**Table 5:** League table for EVB rates.

Carv+EVBL															
0.75 (0.13, 4.42)	FZHYJN+PPL														
0.76 (0.10, 5.64)	1.02 (0.14, 7.31)	Mido+PPL													
0.73 (0.04, 14.48)	0.98 (0.05, 19.88)	0.96 (0.04, 22.30)	PPL+EVBL												
0.36 (0.07, 1.81)	0.49 (0.14, 1.71)	0.48 (0.08, 2.94)	0.50 (0.03, 9.13)	FZHYJN											
0.33 (0.10, 1.15)	0.45 (0.12, 1.63)	0.44 (0.09, 2.14)	0.45 (0.03, 6.87)	0.92 (0.32, 2.63)	EVBL										
0.31 (0.09, 1.07)	0.42 (0.10, 1.66)	0.41 (0.08, 2.14)	0.42 (0.03, 6.85)	0.85 (0.27, 2.74)	0.93 (0.51, 1.71)	Carv									
0.26 (0.05, 1.40)	0.35 (0.06, 1.90)	0.34 (0.05, 2.35)	0.35 (0.02, 6.74)	0.71 (0.16, 3.23)	0.77 (0.24, 2.47)	0.83 (0.23, 3.02)	Nado+ISDM								
0.23 (0.06, 0.93)	0.31 (0.08, 1.23)	0.30 (0.06, 1.60)	0.31 (0.02, 5.12)	0.63 (0.20, 1.98)	0.69 (0.35, 1.36)	0.73 (0.31, 1.76)	0.88 (0.25, 3.11)	EIS							
0.19 (0.05, 0.70)	0.26 (0.07, 0.88)	0.25 (0.05, 1.16)	0.26 (0.02, 4.10)	0.53 (0.20, 1.42)	0.58 (0.38, 0.89)	0.62 (0.33, 1.18)	0.75 (0.23, 2.46)	0.84 (0.42, 1.69)	PPL						
0.14 (0.01, 2.92)	0.19 (0.01, 3.86)	0.19 (0.01, 4.37)	0.20 (0.00, 9.39)	0.40 (0.02, 7.12)	0.43 (0.03, 6.77)	0.46 (0.03, 7.64)	0.56 (0.03, 10.51)	0.63 (0.04, 9.86)	0.75 (0.05, 11.67)	Simv					
0.13 (0.01, 1.59)	0.17 (0.01, 2.10)	0.16 (0.01, 2.44)	0.17 (0.01, 5.74)	0.35 (0.03, 3.79)	0.38 (0.04, 3.50)	0.40 (0.04, 3.99)	0.49 (0.04, 5.65)	0.55 (0.06, 5.09)	0.65 (0.07, 6.02)	0.87 (0.03, 27.29)	Timo				
0.11 (0.01, 0.92)	0.15 (0.02, 1.23)	0.14 (0.01, 1.47)	0.15 (0.01, 3.75)	0.30 (0.04, 2.19)	0.33 (0.06, 1.88)	0.35 (0.06, 2.20)	0.42 (0.07, 2.57)	0.48 (0.08, 2.87)	0.56 (0.10, 3.30)	0.75 (0.03, 18.37)	0.86 (0.05, 13.67)	Nado+EVBL			
0.12 (0.03, 0.55)	0.16 (0.04, 0.74)	0.16 (0.03, 0.95)	0.17 (0.01, 2.90)	0.34 (0.09, 1.22)	0.37 (0.15, 0.88)	0.39 (0.14, 1.11)	0.47 (0.18, 1.27)	0.54 (0.20, 1.41)	0.64 (0.26, 1.57)	0.85 (0.05, 14.14)	0.97 (0.10, 9.80)	1.13 (0.25, 5.15)	Nado		
0.12 (0.02, 0.58)	0.16 (0.03, 0.76)	0.15 (0.02, 0.94)	0.16 (0.01, 2.92)	0.32 (0.08, 1.29)	0.35 (0.12, 0.99)	0.38 (0.12, 1.19)	0.45 (0.10, 2.09)	0.51 (0.16, 1.68)	0.61 (0.23, 1.62)	0.81 (0.04, 14.84)	0.93 (0.08, 10.49)	1.08 (0.14, 8.06)	0.96 (0.26, 3.57)	ISDN	
0.08 (0.02, 0.31)	0.11 (0.03, 0.40)	0.11 (0.02, 0.53)	0.11 (0.01, 1.76)	0.22 (0.08, 0.63)	0.24 (0.14, 0.42)	0.26 (0.12, 0.56)	0.31 (0.10, 1.00)	0.35 (0.20, 0.61)	0.42 (0.24, 0.72)	0.56 (0.04, 8.22)	0.64 (0.07, 5.53)	0.74 (0.13, 4.19)	0.66 (0.29, 1.51)	0.69 (0.23, 2.08)	PBO

##### 3.4.2.3 Consistency assessment

Node-splitting analysis was used to assess consistency; a *P*-value < 0.05 was considered indicative of local inconsistency, and the corresponding contrasts were not pooled. As reported in **Table 6**, every node-split comparison yielded *P >*0.05, demonstrating coherent direct and indirect estimates throughout the network.

**Table 6:** Node-splitting test table for EVB rates.

Side	Direct		Indirect		Difference		*P*>z
	Coef.	Std. err.	Coef.	Std. err.	Coef.	Std. err.	
PPL vs Carv	-0.25349	0.449731	-0.75372	0.494243	0.500234	0.668212	0.454
PPL vs ISDN	0.435672	0.53365	1.109334	1.723915	-0.67366	1.814307	0.71
PPL vs PBO	0.736552	0.364342	1.055278	0.418853	-0.31873	0.548853	0.561
PPL vs FZHYJN	-0.43563	0.596442	-1.14054	0.945373	0.704901	1.1179	0.528
PPL vs EIS	1.142289	1.011179	-0.35306	0.373977	1.495345	1.079074	0.166
PPL vs EVBL	-0.53606	0.262719	-0.57956	0.422472	0.043505	0.496793	0.93
PPL vs FZHYJN+PPL	-1.26242	0.648923	-2.67222	2.17877	1.409802	2.2358	0.528
Carv vs EVBL	0.001297	0.362092	-0.29249	0.655977	0.293783	0.751725	0.696
Carv vs Carv+EVBL	-1.33022	0.7105	-0.50037	1.396718	-0.82985	1.537081	0.589
Nado vs PBO	0.451079	0.52265	0.364182	0.748768	0.086897	0.911879	0.924
Nado vs EVBL	-0.68088	0.853702	-1.12368	0.531091	0.442801	1.005418	0.66
Nado vs Nado+EVBL	0.12189	0.774512	-0.90735	1844.374	1.029236	1844.374	1
Nado vs Nado+ISDM	-0.92288	0.571781	-0.09912	1.088093	-0.82376	1.229217	0.503
Timo vs PBO	0.443931	1.099213	1.774825	4050.56	-1.33089	4050.56	1
ISDN vs EVBL	-1.39284	0.891399	-0.81566	0.7148	-0.57718	1.184932	0.626
PBO vs Simv	-0.58345	1.372289	-2.8916	4604.252	2.308154	4604.252	1
PBO vs FZHYJN	-1.96612	0.901343	-1.26122	0.661266	-0.7049	1.1179	0.528
PBO vs EIS	-1.08614	0.305788	-0.76251	0.76679	-0.32363	0.823177	0.694
PBO vs EVBL	-1.44198	0.524956	-1.41584	0.34994	-0.02614	0.630708	0.967
FZHYJN vs FZHYJN+PPL	-0.82678	0.663576	0.58302	2.165315	-1.40981	2.235805	0.528
EIS vs EVBL	-0.09201	0.543865	-0.58697	0.461971	0.494955	0.713512	0.488
EVBL vs PPL+EVBL	-0.78846	1.385401	0.781796	3021.606	-1.57025	3021.606	1
EVBL vs Carv+EVBL	-0.93525	0.715538	-1.76509	1.388981	0.829844	1.537081	0.589
EVBL vs Nado+ISDM	0.770108	0.971369	-0.05365	0.753271	0.823761	1.229217	0.503

#### 3.4.3 All-cause mortality rate

##### 3.4.3.1 Network geometry

**Figure 8:**
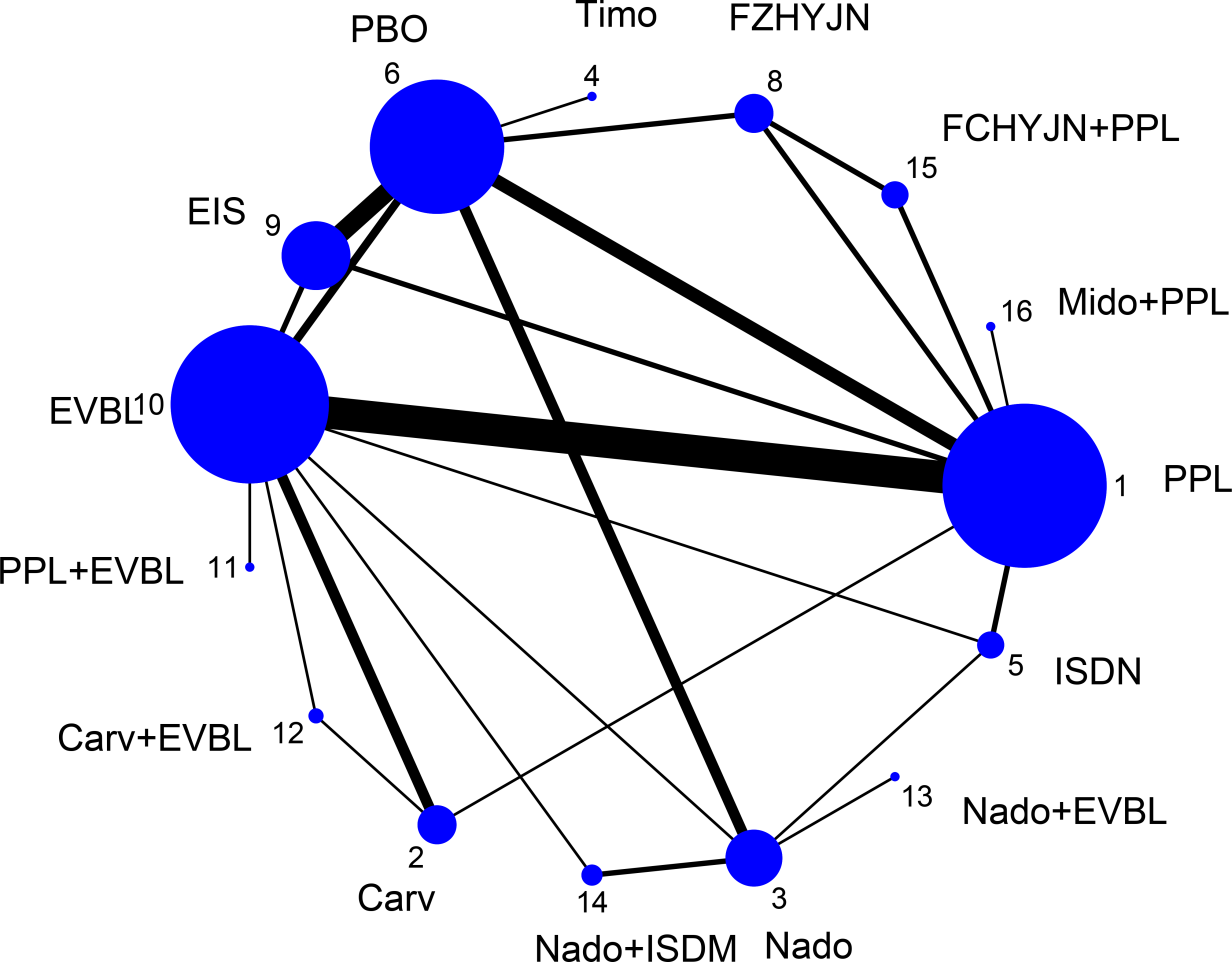
All-cause mortality rate network graph.

All-cause mortality was defined as the incidence of death from any etiology occurring within the predefined observation period, irrespective of whether the fatal event was attributable to disease progression, severe adverse reactions, accident, or any other cause. 50 trials (2 single-arm, 45 two-arm and 3 three-arm) enrolling 5671 participants and evaluating 15 preventive strategies reported this outcome; the resulting network was displayed in **Figure 8**.

##### 3.4.3.2 Network meta-analysis results and probability rankings

**Figure 9:**
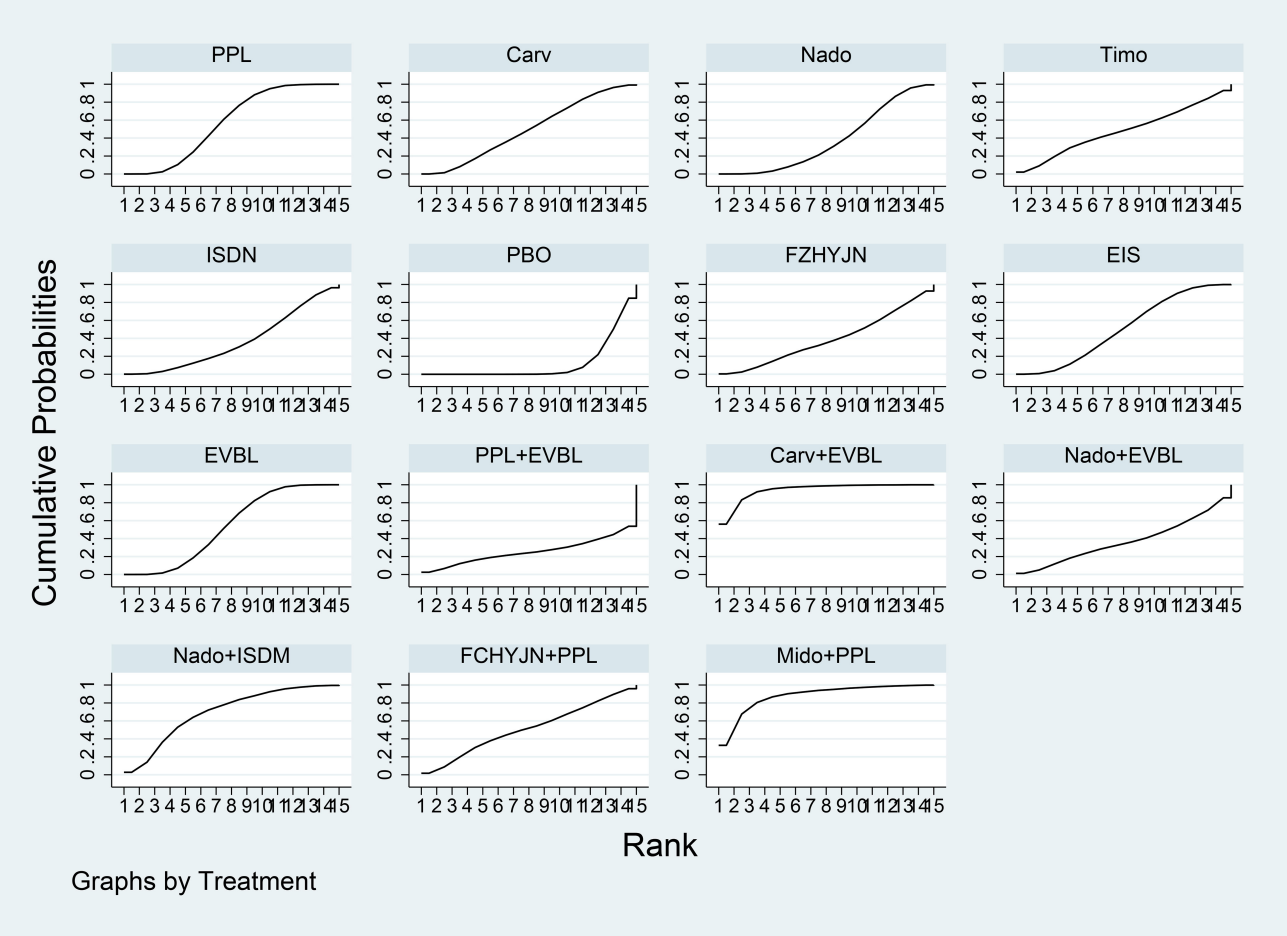
All-cause mortality rate SUCRA plot.

Network meta-analysis results were presented in **Table 7**. All interventions demonstrated statistically significant superiority over placebo. Compared with placebo, Carv+EVBL, Mido+PPL, and Nado+ISDM significantly reduced all-cause mortality with statistical significance, among which Carv+EVBL yielded the lowest all-cause mortality in primary prevention. Regarding NSBB, the ranking was PPL > Carv > Timo > Nado, with statistically significant differences. The cumulative probability ranking plot (**Figure 9**) indicated that a larger area under the curve corresponds to a higher probability of benefit for the corresponding outcome. The ranking of interventions based on the probability of reducing all-cause mortality was as follows: Carv+EVBL > Mido+PPL > Nado+ISDM > PPL > EVBL > FZHYJN+PPL > EIS > Carv > Timo > FZHYJN > Nado > Nado+EVBL > ISDN >PPL+EVBL > PBO.

**Table 7:** League table for all-cause mortality rate.

Carv+EVBL														
0.78 (0.17, 3.63)	Mido+PPL													
0.43 (0.12, 1.53)	0.55 (0.14, 2.10)	Nado+ISDM												
0.34 (0.12, 0.99)	0.44 (0.14, 1.31)	0.79 (0.37, 1.71)	PPL											
0.33 (0.12, 0.92)	0.42 (0.13, 1.32)	0.77 (0.36, 1.63)	0.97 (0.72, 1.31)	EVBL										
0.33 (0.08, 1.36)	0.42 (0.10, 1.79)	0.76 (0.23, 2.57)	0.96 (0.37, 2.48)	0.99 (0.37, 2.66)	FZHYJN+PPL									
0.32 (0.10, 0.98)	0.41 (0.12, 1.34)	0.74 (0.33, 1.68)	0.94 (0.61, 1.45)	0.97 (0.62, 1.51)	0.97 (0.35, 2.73)	EIS								
0.32 (0.12, 0.89)	0.41 (0.12, 1.44)	0.75 (0.30, 1.87)	0.94 (0.52, 1.72)	0.97 (0.57, 1.66)	0.98 (0.32, 2.98)	1.00 (0.50, 2.00)	Carv							
0.31 (0.07, 1.39)	0.39 (0.08, 1.85)	0.72 (0.20, 2.59)	0.90 (0.30, 2.69)	0.93 (0.31, 2.80)	0.94 (0.22, 3.93)	0.96 (0.32, 2.89)	0.96 (0.28, 3.25)	Timo						
0.27 (0.07, 1.05)	0.34 (0.08, 1.39)	0.62 (0.20, 1.95)	0.79 (0.33, 1.86)	0.81 (0.33, 2.00)	0.82 (0.31, 2.18)	0.84 (0.33, 2.14)	0.84 (0.30, 2.36)	0.87 (0.22, 3.42)	FZHYJN					
0.28 (0.09, 0.88)	0.35 (0.10, 1.21)	0.65 (0.34, 1.21)	0.81 (0.47, 1.40)	0.84 (0.49, 1.44)	0.84 (0.29, 2.49)	0.87 (0.48, 1.58)	0.86 (0.41, 1.83)	0.90 (0.28, 2.86)	1.03 (0.38, 2.80)	Nado				
0.25 (0.06, 1.18)	0.33 (0.07, 1.59)	0.59 (0.18, 1.93)	0.75 (0.24, 2.33)	0.77 (0.25, 2.40)	0.78 (0.18, 3.38)	0.80 (0.25, 2.55)	0.79 (0.23, 2.77)	0.83 (0.18, 3.81)	0.95 (0.23, 3.89)	0.92 (0.34, 2.50)	Nado+EVBL			
0.27 (0.08, 0.88)	0.34 (0.10, 1.19)	0.62 (0.25, 1.54)	0.78 (0.43, 1.42)	0.81 (0.43, 1.50)	0.81 (0.27, 2.47)	0.83 (0.41, 1.70)	0.83 (0.37, 1.87)	0.87 (0.25, 2.95)	0.99 (0.35, 2.81)	0.96 (0.47, 1.98)	1.05 (0.31, 3.58)	ISDN		
0.16 (0.02, 1.39)	0.21 (0.02, 1.89)	0.38 (0.05, 2.89)	0.48 (0.07, 3.22)	0.50 (0.08, 3.24)	0.50 (0.06, 4.16)	0.52 (0.08, 3.53)	0.51 (0.07, 3.60)	0.54 (0.06, 4.70)	0.62 (0.08, 4.90)	0.60 (0.09, 4.17)	0.65 (0.07, 5.77)	0.62 (0.09, 4.45)	PPL+EVBL	
0.19 (0.06, 0.56)	0.24 (0.08, 0.76)	0.44 (0.21, 0.93)	0.55 (0.40, 0.77)	0.57 (0.40, 0.82)	0.57 (0.21, 1.54)	0.59 (0.41, 0.85)	0.59 (0.31, 1.11)	0.61 (0.22, 1.73)	0.70 (0.29, 1.70)	0.68 (0.41, 1.13)	0.74 (0.24, 2.26)	0.71 (0.37, 1.35)	1.14 (0.17, 7.66)	PBO

##### 3.4.3.3 Consistency assessment

Node-splitting analysis was used to assess consistency; *P*-value < 0.05 was considered indicative of local inconsistency, and the corresponding contrasts were not pooled. As reported in **Table 8** every node-split comparison yielded *P >*0.05, demonstrating coherent direct and indirect estimates throughout the network.

**Table 8:** Node-splitting test table for all-cause mortality rate.

Side	Direct		Indirect		Difference		*P*>z
	Coef.	Std. err.	Coef.	Std. err.	Coef.	Std. err.	
PPL vs Carv	-0.20333	0.779097	0.106132	0.333743	-0.30946	0.845054	0.714
PPL vs ISDN	0.512229	0.349303	-0.51798	0.601315	1.030213	0.702648	0.143
PPL vs PBO	0.435655	0.21264	0.851732	0.273976	-0.41608	0.346583	0.23
PPL vs FZHYJN	0.575838	0.506801	-0.05983	0.248679	0.635665	0.564711	0.26
PPL vs EIS	0.142723	0.177331	-0.29435	0.302031	0.437078	0.350173	0.212
PPL vs Nado+ISDM	-0.11573	0.498182	2.272443	1.886638	-2.38817	1.94859	0.22
Carv vs EIS	0.007279	0.276354	-1.06645	1.314027	1.073732	1.328754	0.419
Carv vs PPL+EVBL	-1.30252	0.554211	-0.24052	1.11331	-1.06201	1.164094	0.362
Nado vs ISDN	-0.29605	0.689077	0.174959	0.440185	-0.47101	0.817673	0.565
Nado vs PBO	0.334067	0.324343	0.480718	0.43492	-0.14665	0.543126	0.787
Nado VS EIS	0.112987	0.568399	-0.26356	0.315898	0.376544	0.650283	0.563
Nado vs Carv+EVBL	0.082888	0.509191	-0.41536	152.3363	0.498244	152.3372	0.997
Nado vs Nado+EVBL	-0.39769	0.360895	-0.6179	0.750636	0.220204	0.833129	0.792
Timo vs PBO	0.490623	0.530462	1.223615	196.4542	-0.73299	196.4547	0.997
ISDN vs EIS	0.142918	0.524862	-0.42804	0.405014	0.570958	0.667875	0.393
PBO vs FZHYJN	-0.64457	0.198078	0.092182	0.453433	-0.73675	0.494034	0.136
PBO vs EIS	-0.82328	0.338798	-0.45042	0.220367	-0.37286	0.403495	0.355
FZHYJN vs EIS	-0.22762	0.374806	0.084709	0.288432	-0.31233	0.472721	0.509
EIS vs EVBL	0.693147	0.953978	-0.18035	220.038	0.873502	220.0412	0.997
EIS vs PPL+EVBL	-0.91811	0.568744	-1.98009	1.091093	1.061979	1.164087	0.362
EIS vs Nado+EVBL	-0.41552	0.689777	-0.1953	0.467256	-0.22022	0.833138	0.792

#### 3.4.4 Bleeding-related mortality rate

##### 3.4.4.1 Network geometry

**Figure 10:**
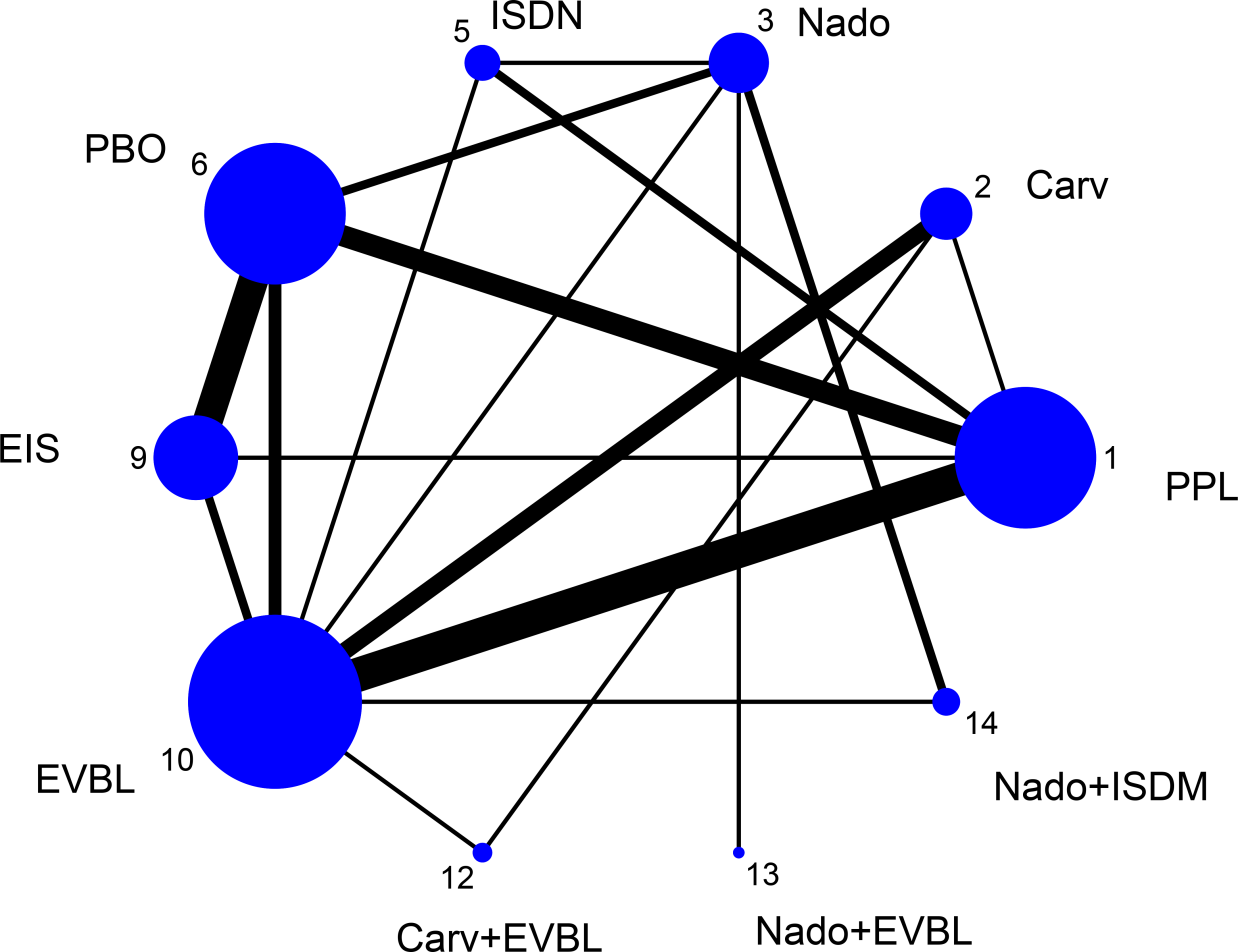
Bleeding-related mortality rate network graph.

The term “mortality” in this context specifically refers to deaths directly caused by variceal bleeding during pharmacological prophylaxis, excluding those resulting from other causes (such as non-hemorrhagic complications, accidents, or other diseases). A total of 40 studies reported outcomes related to all-cause bleeding rates, including 5 single-arm studies, 31 two-arm studies, and 4 three-arm studies. These studies involved 4588 patients and 10 prophylactic interventions. The network diagram was presented in **Figure 10**.

##### 3.4.4.2 Network meta-analysis results and probability rankings

**Figure 11:**
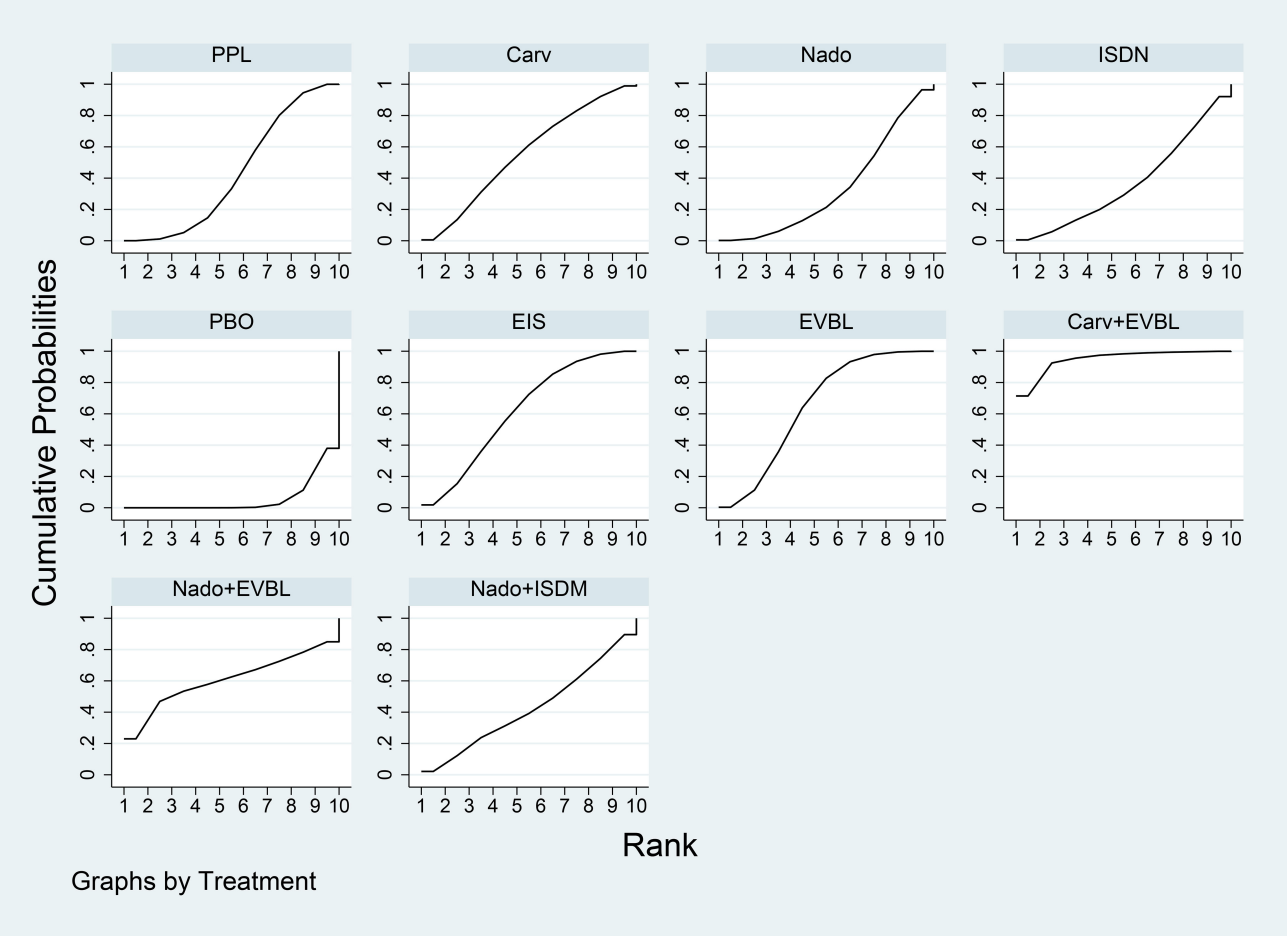
Bleeding-related mortality rate SUCRA plot.

As detailed in **Table 9**, the network meta-analysis indicated that all included interventions were statistically significantly superior to placebo. Specifically, Carv+EVBL, EVBL, and EIS each demonstrated a marked reduction in bleeding-related mortality compared to placebo, with Carv+EVBL associated with the lowest incidence of bleeding-related mortality in the context of primary prevention. With regard to NSBB outcomes, a statistically significant hierarchy was observed: Carv > PPL > Nado. The cumulative probability ranking diagram (**Figure 11**) illustrated that a larger area under the curve corresponded to a higher probability of clinical benefit for the corresponding outcome measure. The ranking of interventions based on their probability of reducing bleeding-related mortality was as follows: Carv+EVBL > EVBL > EIS > Nado+EVBL > Carv > Nado+ISDM > PPL > ISDN > Nado > PBO.

**Table 9:** League table for bleeding-related mortality rate.

Carv+EVBL									
0.27 (0.07, 1.06)	EVBL								
0.26 (0.05, 1.22)	0.96 (0.47, 1.97)	EIS							
0.32 (0.02, 6.58)	1.23 (0.09, 17.65)	1.27 (0.09, 18.74)	Nado+EVBL						
0.24 (0.06, 0.94)	0.90 (0.42, 1.94)	0.94 (0.33, 2.69)	0.74 (0.05, 11.85)	Carv					
0.18 (0.03, 1.32)	0.70 (0.17, 2.80)	0.72 (0.17, 3.13)	0.57 (0.04, 9.06)	0.77 (0.16, 3.77)	Nado+ISDN				
0.19 (0.04, 0.86)	0.73 (0.43, 1.25)	0.76 (0.38, 1.51)	0.60 (0.04, 8.53)	0.81 (0.32, 2.04)	1.05 (0.26, 4.27)	PPL			
0.16 (0.03, 0.95)	0.62 (0.22, 1.79)	0.65 (0.21, 2.02)	0.51 (0.03, 8.13)	0.69 (0.19, 2.55)	0.89 (0.18, 4.53)	0.85 (0.33, 2.21)	ISDN		
0.16 (0.03, 0.87)	0.60 (0.23, 1.57)	0.63 (0.23, 1.73)	0.49 (0.04, 5.94)	0.67 (0.20, 2.27)	0.87 (0.26, 2.91)	0.83 (0.32, 2.12)	0.97 (0.29, 3.29)	Nado	
0.09 (0.02, 0.39)	0.33 (0.18, 0.59)	0.34 (0.20, 0.60)	0.27 (0.02, 3.77)	0.36 (0.14, 0.95)	0.47 (0.12, 1.88)	0.45 (0.28, 0.73)	0.53 (0.19, 1.49)	0.55 (0.23, 1.31)	PBO

##### 3.4.4.3 Consistency assessment

Consistency was evaluated using the node-splitting method. A *P*-value below 0.05 was considered indicative of significant local inconsistency, in which case the corresponding data subset was excluded from pooling. As presented in **Table 10**, all *P*-values for the relevant outcome measures exceeded 0.05, demonstrating good consistency across compared nodes and absence of significant inconsistency.

**Table 10:** Node-splitting test table for bleeding-related mortality rate.

Side	Direct		Indirect		Difference		*P*>z
	Coef.	Std. err.	Coef.	Std. err.	Coef.	Std. err.	
PPL vs Carv	-0.09016	1.267186	-0.24469	0.513773	0.154528	1.34561	0.909
PPL vs Timo	-0.14714	0.52271	1.701663	1.142357	-1.8488	1.242546	0.137
PPL vs ISDN	0.710331	0.284981	1.045131	0.502208	-0.3348	0.573981	0.56
PPL vs PBO	0.675673	1.249181	-0.36256	0.369268	1.038235	1.305495	0.426
PPL vs EVBL	-0.08597	0.328613	-0.74476	0.444994	0.65879	0.545897	0.228
Carv vs EVBL	-0.07329	0.381761	-2.66602	1.859568	2.592737	1.845232	0.16
Carv vs FZHYJN	-1.56446	0.735164	-0.3397	1.559475	-1.22475	1.568928	0.435
Nado vs ISDN	1.428841	1.189223	-0.57429	0.729803	2.003134	1.395301	0.151
Nado vs PBO	0.581367	0.555388	0.650535	0.803365	-0.06917	0.976624	0.944
Nado vs EVBL	-1.14028	1.213105	-0.38099	0.53825	-0.7593	1.327155	0.567
Nado vs Nado+EVBL	-0.70775	1.270408	-0.4958	436.8491	-0.21195	436.8498	1
Nado vs Nado+ISDM	-0.39637	0.701393	0.769416	1.33093	-1.16578	1.504382	0.438
ISDN vs EVBL	-0.08267	1.253656	-0.58511	0.628541	0.502444	1.426948	0.725
PBO vs EIS	-1.22312	0.281869	-0.04849	0.722374	-1.17462	0.764653	0.125
PBO vs EVBL	-1.05752	0.526956	-1.14071	0.379724	0.083189	0.649805	0.898
EIS vs EVBL	-0.31389	0.541856	0.186165	0.497076	-0.50005	0.736007	0.497
EVBL vs Carv+EVBL	-1.15632	0.75779	-2.381	1.526622	1.224679	1.568912	0.435
EVBL vs Nado+ISDM	1.133707	1.22358	-0.03212	0.875269	1.165831	1.504413	0.438

#### 3.4.5 Incidence of general adverse events rate

##### 3.4.5.1 Network geometry

**Figure 12:**
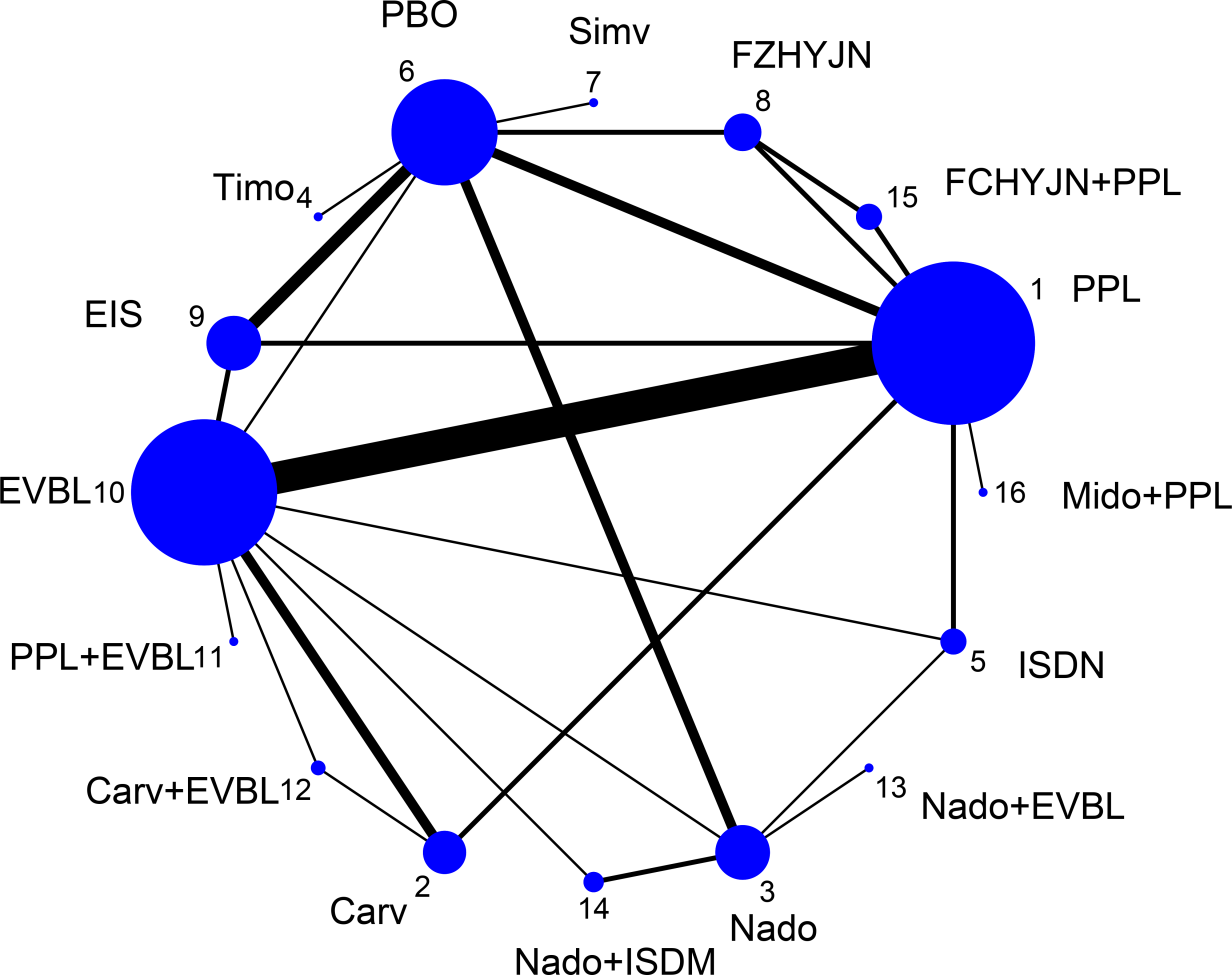
Incidence of general adverse events rate network graph.

Adverse events under this definition refer to non-life-threatening, non-serious reactions occurring during pharmacological treatment, commonly including symptoms such as cough, vomiting, and decreased heart rate. A total of 52 studies reported outcomes related to general adverse events, comprising 3 single-arm studies, 44 two-arm studies, and 5 three-arm studies. The analysis involved 6044 patients and 16 prophylactic interventions. The network diagram was presented in **Figure 12**.

##### 3.4.5.2 Network meta-analysis results and probability rankings

**Figure 13:**
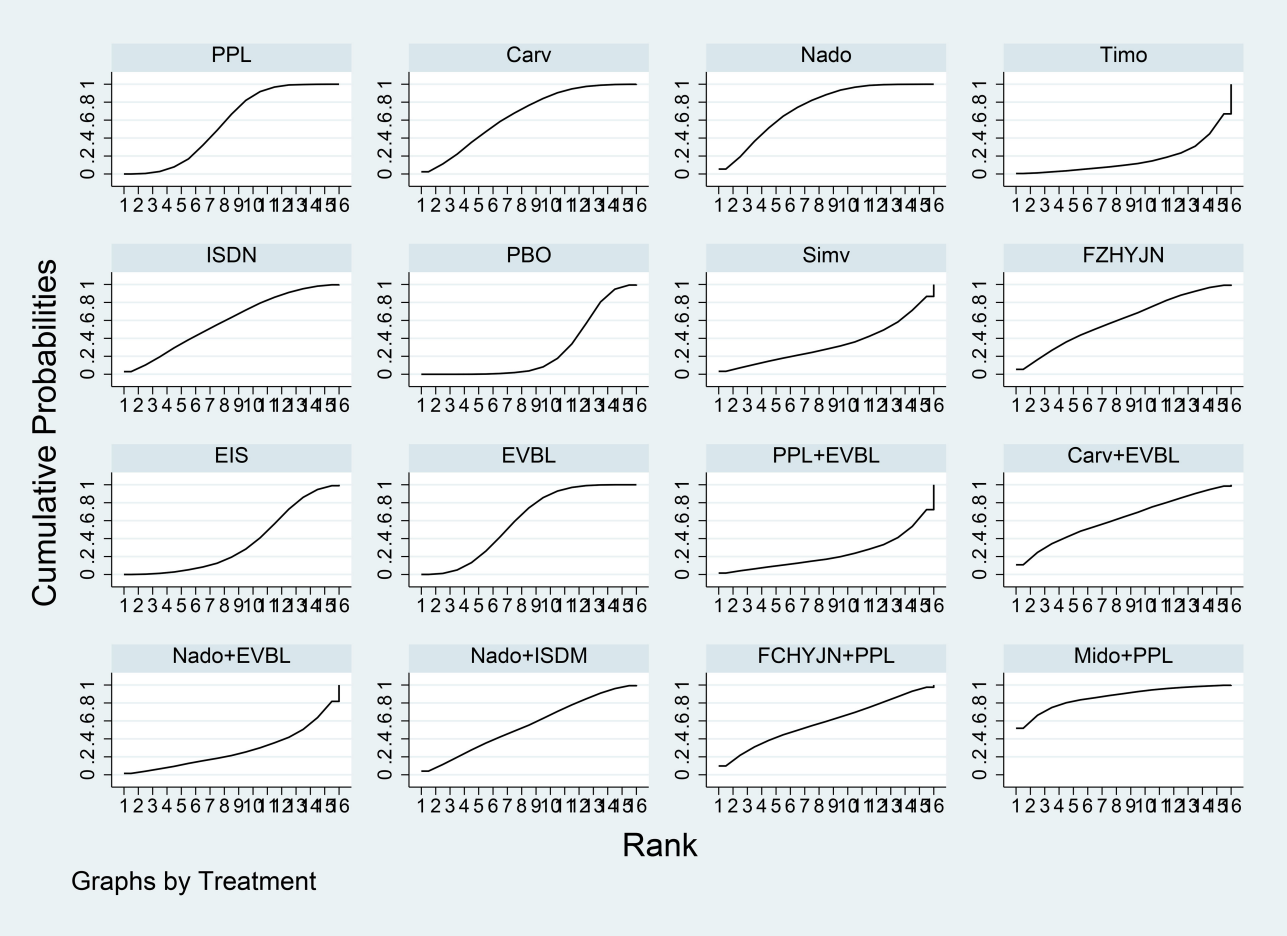
Incidence of general adverse events Rate SUCRA Plot.

Results of the network meta-analysis were presented in **Table 11**. All interventions demonstrated statistically significant superiority over Timo. The inferior performance of Timo compared to placebo may be attributed to the following reasons: (1) Patients with cirrhosis inherently exhibited relative effective arterial hypovolemia. Timo, by reducing cardiac output, can exacerbate this condition, leading to a decline in arterial blood pressure (108, 109). (2) In patients with decompensated cirrhosis (Child-Pugh class B or C), the reduction in cardiac output and risk of hypotension induced by Timo may counteract or even outweigh its potential benefits in reducing portal pressure and preventing variceal bleeding (110). Mido+PPL, Nado, and Carv were associated with lower incidence rates of general adverse events and demonstrated favourable efficacy, with Mido+PPL showing the lowest incidence of general adverse events in primary prevention. For NSBB outcomes, the hierarchy was Nado > Carv > PPL > Timo, with statistically significant differences. The cumulative ranking probability curve (**Figure 13**) indicated that a larger area under the curve corresponded to a greater probability of benefit for the respective outcome. The ranking of interventions based on the probability of reducing bleeding-related mortality was as follows: Mido+PPL > Nado > Carv > Carv+EVBL > FZHYJN > EVBL > ISDN > FZHYJN+PPL > PPL > Nado+ISDM > EIS > Simv > Nado+EVBL > PBO > PPL+EVBL > Timo.

**Table 11:** League table for incidence of general adverse events rate.

Mido+PPL															
0.49 (0.07, 3.53)	Nado														
0.41 (0.06, 2.81)	0.84 (0.26, 2.75)	Carv													
0.42 (0.04, 4.33)	0.85 (0.14, 5.02)	1.01 (0.22, 4.62)	Carv+EVBL												
0.37 (0.04, 3.33)	0.75 (0.16, 3.64)	0.90 (0.19, 4.31)	0.89 (0.11, 6.94)	FZHYJN											
0.36 (0.06, 2.16)	0.73 (0.28, 1.87)	0.86 (0.40, 1.87)	0.85 (0.19, 3.89)	0.96 (0.23, 3.97)	EVBL										
0.36 (0.05, 2.72)	0.73 (0.21, 2.53)	0.87 (0.24, 3.18)	0.86 (0.14, 5.49)	0.97 (0.18, 5.27)	1.01 (0.34, 3.00)	ISDN									
0.37 (0.03, 4.19)	0.76 (0.11, 5.11)	0.90 (0.14, 5.90)	0.89 (0.09, 8.90)	1.00 (0.18, 5.46)	1.04 (0.18, 6.02)	1.03 (0.14, 7.48)	FZHYJN+PPL								
0.34 (0.06, 1.93)	0.70 (0.27, 1.78)	0.83 (0.36, 1.88)	0.82 (0.17, 3.92)	0.92 (0.24, 3.56)	0.96 (0.59, 1.55)	0.95 (0.33, 2.69)	0.92 (0.17, 5.01)	PPL							
0.34 (0.04, 3.06)	0.69 (0.22, 2.22)	0.82 (0.18, 3.76)	0.81 (0.11, 6.07)	0.92 (0.14, 5.91)	0.95 (0.25, 3.61)	0.94 (0.19, 4.66)	0.91 (0.11, 7.84)	1.00 (0.26, 3.85)	Nado+ISDM						
0.22 (0.03, 1.50)	0.45 (0.15, 1.31)	0.54 (0.18, 1.60)	0.53 (0.09, 2.94)	0.60 (0.13, 2.69)	0.62 (0.27, 1.42)	0.61 (0.17, 2.19)	0.60 (0.09, 3.79)	0.65 (0.29, 1.46)	0.65 (0.15, 2.82)	EIS					
0.18 (0.01, 2.45)	0.37 (0.05, 2.80)	0.44 (0.05, 3.55)	0.43 (0.04, 5.15)	0.49 (0.05, 4.86)	0.51 (0.07, 3.64)	0.50 (0.06, 4.42)	0.49 (0.04, 6.23)	0.53 (0.08, 3.72)	0.53 (0.05, 5.18)	0.82 (0.11, 5.91)	Simv				
0.15 (0.01, 2.07)	0.31 (0.05, 1.73)	0.36 (0.04, 2.97)	0.36 (0.03, 4.29)	0.41 (0.04, 4.22)	0.42 (0.06, 3.03)	0.42 (0.05, 3.50)	0.40 (0.03, 5.33)	0.44 (0.06, 3.16)	0.44 (0.05, 3.57)	0.68 (0.09, 5.21)	0.83 (0.06, 12.01)	Nado+EVBL			
0.19 (0.03, 1.19)	0.38 (0.16, 0.90)	0.45 (0.16, 1.23)	0.44 (0.08, 2.34)	0.50 (0.13, 2.00)	0.52 (0.25, 1.07)	0.52 (0.16, 1.65)	0.50 (0.09, 2.94)	0.54 (0.28, 1.05)	0.55 (0.14, 2.09)	0.84 (0.40, 1.75)	1.03 (0.16, 6.48)	1.24 (0.18, 8.60)	PBO		
0.12 (0.01, 1.72)	0.24 (0.03, 2.17)	0.28 (0.03, 2.40)	0.28 (0.02, 3.43)	0.32 (0.03, 3.65)	0.33 (0.04, 2.41)	0.33 (0.03, 3.15)	0.32 (0.02, 4.50)	0.34 (0.04, 2.67)	0.35 (0.03, 3.79)	0.53 (0.06, 4.59)	0.65 (0.04, 10.75)	0.78 (0.05, 12.93)	0.63 (0.08, 5.25)	PPL+EVBL	
0.10 (0.01, 1.17)	0.20 (0.03, 1.30)	0.23 (0.03, 1.65)	0.23 (0.02, 2.45)	0.26 (0.03, 2.29)	0.27 (0.04, 1.68)	0.27 (0.03, 2.06)	0.26 (0.02, 2.97)	0.28 (0.05, 1.71)	0.28 (0.03, 2.43)	0.43 (0.07, 2.72)	0.53 (0.04, 6.43)	0.64 (0.05, 8.33)	0.52 (0.10, 2.78)	0.82 (0.05, 12.20)	Timo

##### 3.4.5.3 Consistency assessment

Consistency was evaluated using the node-splitting approach. *P*-value below 0.05 indicated the presence of local inconsistency, leading to exclusion of the corresponding data from pooling. As shown in **Table 12**, all *P*-values for the relevant outcomes in this study exceeded 0.05, demonstrating good consistency across comparison nodes without significant inconsistency.

**Table 12:** Node-splitting test table for incidence of general adverse events rate.

Side	Direct		Indirect		Difference		*P*>z
	Coef.	Std. err.	Coef.	Std. err.	Coef.	Std. err.	
PPL vs Carv	-0.6472503	0.633276	0.180895	0.570953	-0.82815	0.850891	0.33
PPL vs ISDN	-0.107843	0.641573	0.081282	1.016008	-0.18913	1.200935	0.875
PPL vs PBO	0.7222164	0.452811	0.46763	0.510084	0.254587	0.68177	0.709
PPL vs FZHYJN	-5.62E-12	0.884513	-0.20991	1.116537	0.209907	1.424436	0.883
PPL vs EIS	0.3923091	0.942374	0.448549	0.471551	-0.05624	1.054186	0.957
PPL vs EVBL	-0.1197674	0.282502	0.249291	0.540504	-0.36906	0.607694	0.544
PPL vs FZHYJN+PPL	-0.0449202	0.912143	-0.46473	2.716964	0.419814	2.848872	0.883
Carv vs EVBL	-0.083306	0.45657	0.911421	0.842929	-0.99473	0.965368	0.303
Carv vs Carv+EVBL	0.0453686	0.88968	-0.2427	1.78332	0.288073	1.993388	0.885
Nado vs ISDN	-0.5965213	1.080192	0.773842	0.773683	-1.37036	1.328683	0.302
Nado vs PBO	0.875635	0.540898	1.167673	0.786119	-0.29204	0.954161	0.76
Nado vs EVBL	0.926	1.040331	0.150075	0.548703	0.775925	1.176164	0.509
Nado vs Nado+EVBL	1.185624	0.885344	0.757264	126.9724	0.42836	126.9755	0.997
Nado vs Nado+ISDM	0.6123663	0.682856	-0.44765	1.245715	1.060012	1.420599	0.456
Timo vs PBO	-0.6622114	0.859422	1.246529	128.9208	-1.90874	128.9237	0.988
ISDN vs EVBL	-1.146469	0.91894	0.655061	0.68747	-1.80153	1.159106	0.12
PBO vs Simv	0.029853	0.938242	-1.2141	181.7376	1.243951	181.7399	0.995
PBO vs FZHYJN	-0.8064714	1.061045	-0.59659	0.950379	-0.20988	1.42444	0.883
PBO vs EIS	-0.1688254	0.444278	-0.18143	0.757097	0.012608	0.881115	0.989
PBO vs EVBL	0.5795965	0.994105	-0.84482	0.389324	1.424416	1.068889	0.183
FZHYJN vs FZHYJN+PPL	-0.0449202	0.912143	0.374894	2.717499	-0.41981	2.848872	0.883
EIS vs EVBL	-0.7361776	0.65612	-0.28373	0.569563	-0.45244	0.868944	0.603
EVBL vs PPL+EVBL	1.111231	1.015397	0.214566	147.6014	0.896665	147.6042	0.995
EVBL vs Carv+EVBL	-0.2149145	0.887122	0.073205	1.787098	-0.28812	1.993353	0.885
EVBL vs Nado+ISDM	-0.6241529	1.131674	0.435924	0.858752	-1.06008	1.420612	0.456

#### 3.4.6 Incidence of other decompensation events rate

##### 3.4.6.1 Network geometry

**Figure 14:**
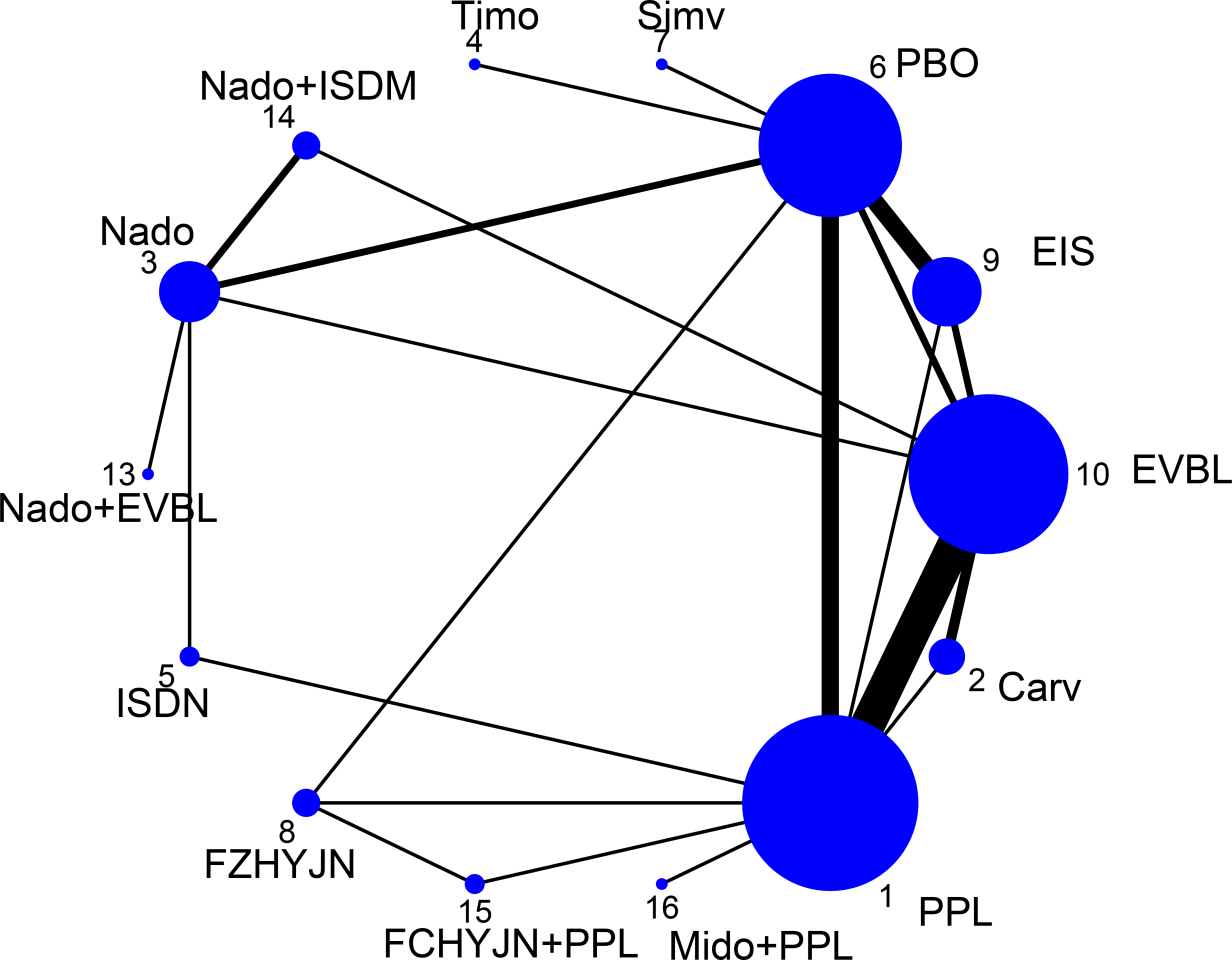
Incidence of other decompensation events rate network graph.

This definition encompasses clinical manifestations related to hepatic decompensation occurring either during primary prophylaxis or in the end-of-life period until death, including complications such as ascites, spontaneous bacterial peritonitis, jaundice, and hepatorenal syndrome. A total of 41 studies reported outcomes related to decompensation events, consisting of 1 single-arm study, 35 two-arm studies, and 3 three-arm studies. The analysis involved 4452 patients and 14 prophylactic interventions. The network diagram was illustrated in **Figure 14**.

##### 3.4.6.2 Network meta-analysis results and probability rankings

As presented in **Table 13**, all interventions demonstrated statistically significant superiority over simvastatin. The relatively poor performance of simvastatin may be attributed to its potential to induce drug-related hepatotoxicity and myotoxicity, particularly in patients with pre-existing hepatic impairment (111, 112). This pharmacological characteristic likely explained why its clinical efficacy was inferior even to placebo in certain comparisons. Compared with placebo, ISDM, Mido+PPL, and Carv significantly reduced the incidence of other decompensation events (all with statistical significance), with ISDM showing the lowest rate of such events.

**Table 13:** League table for incidence of other decompensation events rate.

ISDN													
0.79 (0.21, 3.00)	Mido+PPL												
0.66 (0.17, 2.51)	0.83 (0.29, 2.37)	Carv											
0.57 (0.13, 2.49)	0.72 (0.19, 2.70)	0.87 (0.24, 3.09)	Nado+ISDM										
0.46 (0.10, 2.21)	0.59 (0.16, 2.18)	0.70 (0.19, 2.62)	0.81 (0.18, 3.74)	FZHYJN									
0.45 (0.12, 1.65)	0.57 (0.18, 1.78)	0.69 (0.23, 2.02)	0.79 (0.36, 1.72)	0.97 (0.25, 3.82)	Nado								
0.45 (0.09, 2.34)	0.57 (0.12, 2.62)	0.69 (0.16, 3.01)	0.79 (0.22, 2.84)	0.97 (0.18, 5.34)	1.00 (0.36, 2.76)	Nado+EVBL							
0.40 (0.12, 1.29)	0.50 (0.22, 1.15)	0.60 (0.30, 1.22)	0.70 (0.24, 2.04)	0.86 (0.27, 2.70)	0.88 (0.38, 2.04)	0.88 (0.24, 3.29)	EVBL						
0.38 (0.12, 1.18)	0.49 (0.24, 1.00)	0.58 (0.27, 1.23)	0.67 (0.22, 2.02)	0.83 (0.28, 2.47)	0.85 (0.35, 2.03)	0.85 (0.22, 3.23)	0.96 (0.65, 1.43)	PPL					
0.36 (0.10, 1.28)	0.45 (0.17, 1.18)	0.54 (0.21, 1.38)	0.63 (0.19, 2.09)	0.77 (0.23, 2.60)	0.79 (0.29, 2.15)	0.79 (0.19, 3.29)	0.90 (0.47, 1.73)	0.94 (0.50, 1.75)	EIS				
0.30 (0.05, 1.60)	0.38 (0.09, 1.61)	0.45 (0.10, 1.94)	0.52 (0.10, 2.74)	0.64 (0.17, 2.35)	0.66 (0.14, 3.00)	0.66 (0.11, 4.09)	0.74 (0.20, 2.77)	0.77 (0.22, 2.73)	0.83 (0.21, 3.31)	FZHYJN+PPL			
0.27 (0.06, 1.15)	0.34 (0.10, 1.12)	0.41 (0.12, 1.32)	0.47 (0.12, 1.88)	0.58 (0.14, 2.31)	0.59 (0.18, 1.99)	0.59 (0.12, 2.88)	0.67 (0.25, 1.79)	0.70 (0.27, 1.81)	0.75 (0.27, 2.06)	0.90 (0.19, 4.26)	Timo		
0.28 (0.08, 0.93)	0.36 (0.15, 0.84)	0.43 (0.19, 0.99)	0.49 (0.16, 1.50)	0.61 (0.20, 1.83)	0.62 (0.26, 1.50)	0.62 (0.16, 2.38)	0.71 (0.42, 1.18)	0.74 (0.47, 1.16)	0.79 (0.44, 1.39)	0.95 (0.26, 3.52)	1.05 (0.46, 2.44)	PBO	
0.14 (0.02, 0.97)	0.18 (0.03, 1.02)	0.22 (0.04, 1.21)	0.25 (0.04, 1.62)	0.31 (0.05, 1.99)	0.32 (0.06, 1.80)	0.32 (0.04, 2.37)	0.36 (0.08, 1.75)	0.38 (0.08, 1.79)	0.40 (0.08, 1.98)	0.49 (0.07, 3.53)	0.54 (0.10, 2.98)	0.51 (0.12, 2.27)	Simv

**Figure 15:**
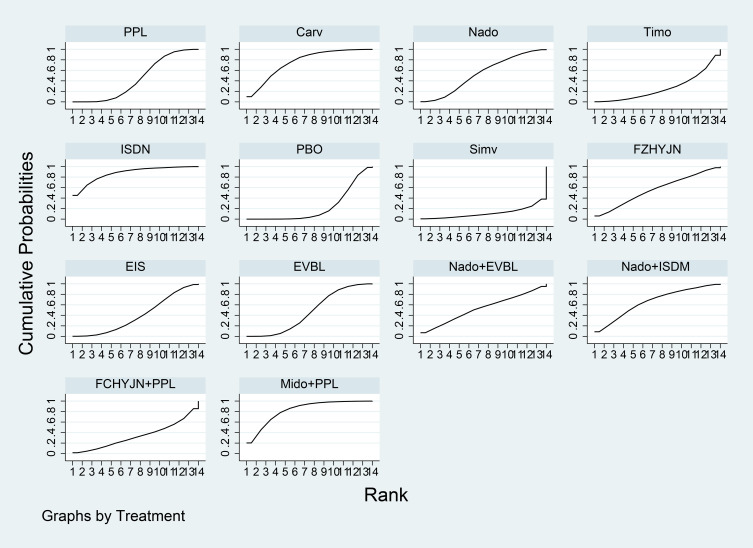
Incidence of other decompensation events rate SUCRA plot.

For the NBSS outcomes, the hierarchy was Nado > PPL > Carv > Timo, demonstrating statistically significant differences. The cumulative ranking probability curve (**Figure 15**) indicated that a larger area under the curve corresponded to a greater likelihood of benefit for the specific outcome. The ranking of interventions based on the probability of preventing bleeding-related mortality was as follows: ISDN > Mido+PPL > Carv > Nado+ISDM > FZHYJN > Nado > Nado+EVBL > EVBL > PPL > EIS > FZHYJN+PPL > Timo > PBO > Simv.

##### 3.4.6.3 Consistency assessment

Consistency was evaluated using the node-splitting method. A *P*-value less than 0.05 was interpreted as evidence of local inconsistency, and data from such comparisons were excluded from the pooled analysis. As presented in **Table 14**, all *P*-values for the outcomes assessed in this study exceeded 0.05, indicating satisfactory consistency across the network and an absence of significant inconsistency.

**Table 14:** Node-splitting test table for incidence of other decompensation events rate.

Side	Direct		Indirect		Difference		*P*>z
	Coef.	Std. err.	Coef.	Std. err.	Coef.	Std. err.	
PPL vs Carv	-0.64523	0.540736	-0.41305	0.558025	-0.23218	0.770825	0.763
PPL vs ISDN	-0.82998	0.644213	-1.49994	1.286611	0.669964	1.438881	0.641
PPL vs PBO	0.435028	0.282094	0.04485	0.404835	0.390178	0.49176	0.428
PPL vs FZHYJN	-0.26236	0.741672	-0.09476	0.862742	-0.1676	1.137717	0.883
PPL vs EIS	-0.39781	0.532269	0.311201	0.389688	-0.70901	0.657256	0.281
PPL vs EVBL	-0.06791	0.231694	0.090929	0.427182	-0.15884	0.481946	0.742
PPL vs FZHYJN+PPL	0.223144	0.685529	0.558348	2.155069	-0.3352	2.275435	0.883
Carv vs EVBL	0.536755	0.390283	0.26845	1.015437	0.268305	1.089999	0.806
Nado vs ISDN	-1.26567	1.197101	-0.5957	0.79833	-0.66996	1.438882	0.641
Nado vs PBO	-0.19638	0.698044	0.920054	0.568316	-1.11644	0.901176	0.215
Nado vs EVBL	0.538997	0.615834	-0.25214	0.590883	0.791138	0.853459	0.354
Nado vs Nado+EVBL	-2.68E-08	0.517816	1.794435	1871.095	-1.79444	1871.095	0.999
Nado vs Nado+ISDM	-0.1305	0.418262	-1.20184	1.275684	1.071343	1.342564	0.425
Timo vs PBO	-0.0526	0.427769	0.540031	1943.13	-0.59263	1943.13	1
PBO vs Simv	0.667172	0.758288	-0.47317	2928.129	1.140344	2928.129	1
PBO vs FZHYJN	-0.41028	0.828788	-0.57789	0.779431	0.167602	1.137718	0.883
PBO vs EIS	-0.11696	0.325773	-0.70182	0.623741	0.584855	0.702842	0.405
PBO vs EVBL	-0.43144	0.528291	-0.31101	0.307485	-0.12044	0.609905	0.843
FZHYJN vs FZHYJN+PPL	0.485508	0.720008	0.150303	2.12107	0.335205	2.275435	0.883
EIS vs EVBL	-0.00669	0.575703	-0.14593	0.412883	0.139245	0.704943	0.843
EVBL vs Nado+ISDM	-1.20397	1.191586	-0.13263	0.618541	-1.07134	1.342561	0.425

### 3.5 Subgroup analysis

#### 3.5.1 All-cause bleeding rate

To determine whether the duration of primary prophylaxis with the study drugs influences outcomes, we stratified the RCTs based on prophylaxis duration for the outcome of all-cause bleeding rates. The studies were divided into three subgroups: less than 12 months, 12–24 months, and more than 24 months. This stratification was performed to evaluate the impact of different prophylaxis durations on bleeding events.

##### 3.5.1.1 1–12 months group

A total of 9 studies were completed within one year, including 7 two-arm studies and 2 three-arm studies. These studies involved 1359 patients and evaluated 9 prophylactic interventions.

**Figure 16:**
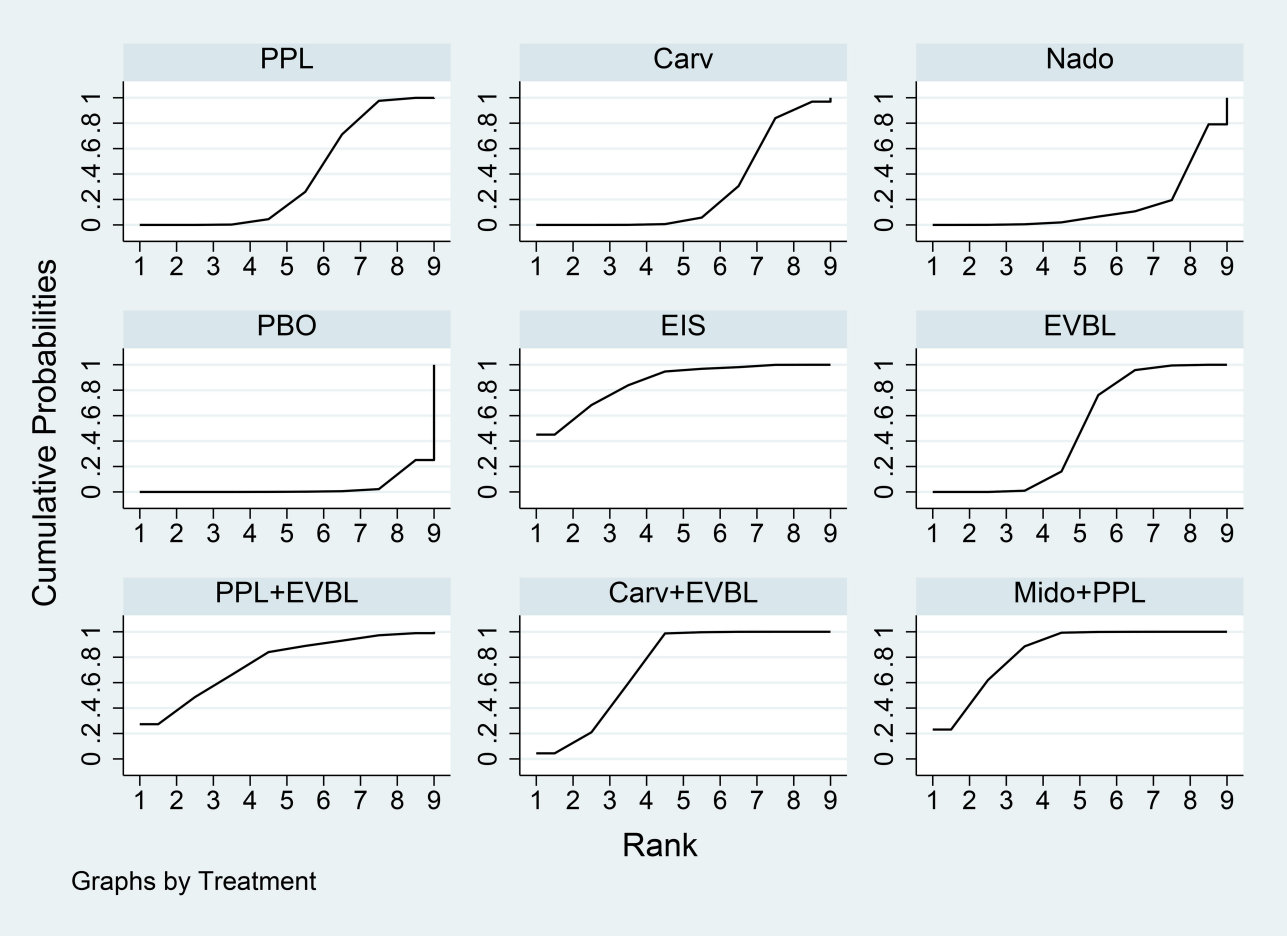
Time Subgroup: All-Cause Bleeding Rates for Months 1-12 SUCRA Plot.

The network meta-analysis results (**Table 15**) indicated that all interventions were statistically significantly superior to placebo. Compared with placebo, EIS, Mido+PPL, and PPL+EVBL were associated with significantly lower all-cause bleeding rates, with EIS demonstrating the lowest rate within the one-year timeframe. For NSBB outcomes, the ranking was PPL > Carv > Nado, with statistically significant differences. The cumulative ranking probability curve (**Figure 16**) showed a larger area under the curve corresponds to a higher probability of benefit for the corresponding outcome. The ranking of interventions based on the probability of preventing bleeding within one year was as follows: EIS > Mido+PPL > PPL+EVBL > Carv+EVBL > EVBL > PPL > Carv > Nado > PBO.

**Table 15:** Time subgroup: league table of all-cause bleeding rates for 1–12 months.

EIS								
0.77 (0.08, 7.14)	Mido+PPL							
0.64 (0.03, 14.42)	0.82 (0.06, 10.93)	PPL+EVBL						
0.46 (0.05, 4.08)	0.60 (0.16, 2.21)	0.72 (0.06, 8.12)	Carv+EVBL					
0.19 (0.02, 1.51)	0.24 (0.08, 0.76)	0.29 (0.03, 2.97)	0.41 (0.20, 0.81)	EVBL				
0.15 (0.02, 1.09)	0.19 (0.07, 0.51)	0.23 (0.02, 2.54)	0.32 (0.13, 0.77)	0.79 (0.44, 1.44)	PPL			
0.12 (0.01, 0.97)	0.15 (0.05, 0.50)	0.19 (0.02, 1.98)	0.26 (0.13, 0.51)	0.63 (0.39, 1.02)	0.80 (0.41, 1.56)	Carv		
0.05 (0.01, 0.35)	0.07 (0.01, 0.42)	0.08 (0.00, 1.41)	0.11 (0.02, 0.68)	0.27 (0.05, 1.49)	0.34 (0.07, 1.68)	0.43 (0.08, 2.41)	Nado	
0.03 (0.01, 0.17)	0.04 (0.01, 0.20)	0.05 (0.00, 0.76)	0.07 (0.02, 0.32)	0.18 (0.05, 0.68)	0.22 (0.07, 0.74)	0.28 (0.07, 1.10)	0.65 (0.23, 1.87)	PBO

##### 3.5.1.2 13–24 months group

**Figure 17:**
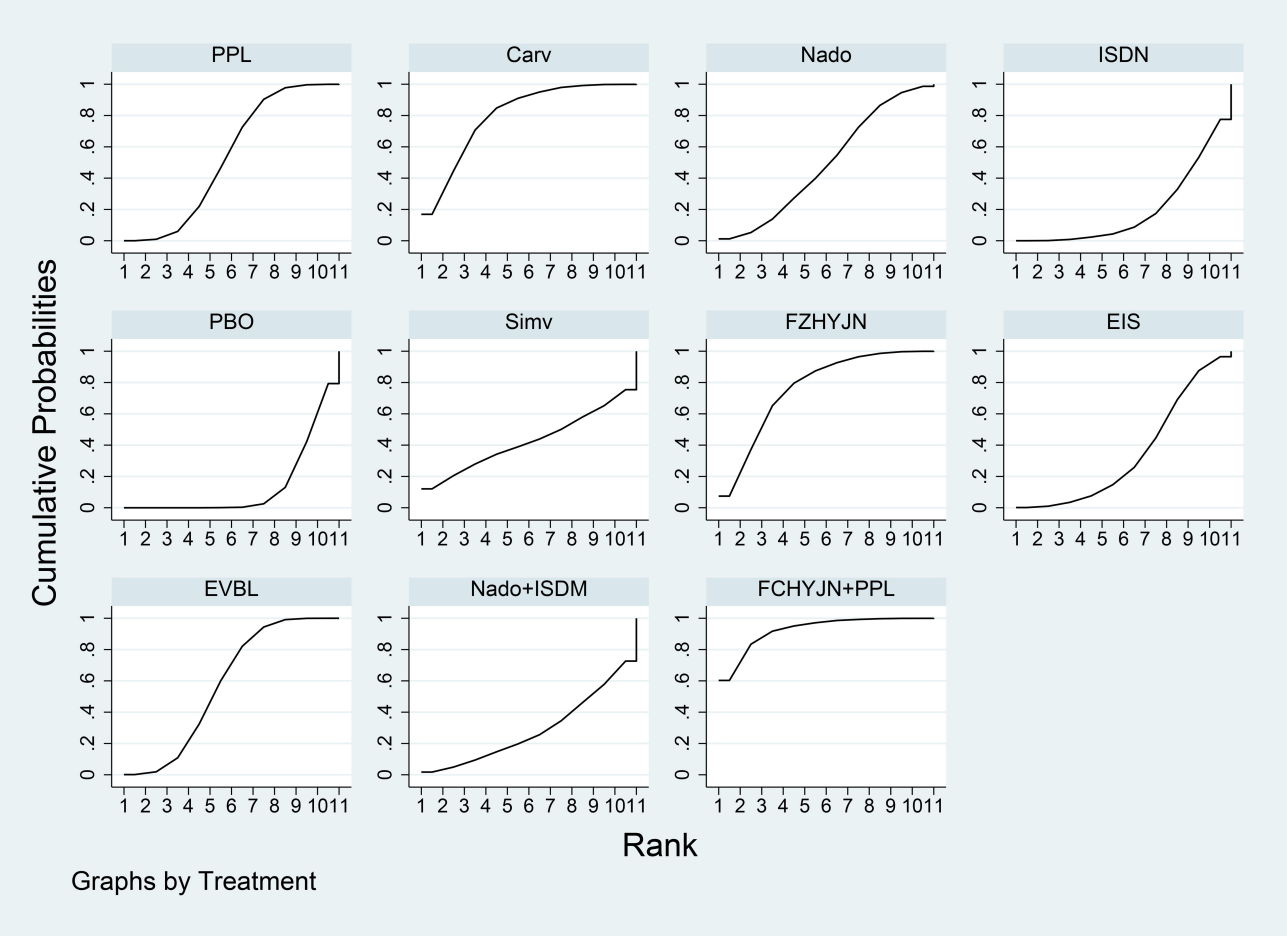
Time subgroup: all-cause bleeding rates for months 13-24 SUCRA plot.

A total of 31 studies were completed within two years, comprising 2 single-arm studies, 24 two-arm studies, and 5 three-arm studies. These studies involved 3355 patients and evaluated 11 prophylactic interventions. The network meta-analysis results (**Table 16**) demonstrated that all interventions were statistically significantly superior to placebo. Compared with placebo, FZHYJN+PPL, Carv, and FZHYJN were associated with significantly lower all-cause bleeding rates, with FZHYJN+PPL showing the lowest rate within the two-year period. For NSBB outcomes, the ranking was Carv > PPL > Nado, with statistically significant differences. The cumulative ranking probability curve (**Figure 17**) indicated that a larger area under the curve corresponds to a higher probability of benefit for the corresponding outcome. The ranking of interventions based on the probability of preventing bleeding within two years was as follows: FZHYJN+PPL > Carv > FZHYJN > EVBL > PPL > Nado > Simv > EIS > Nado+ISDM > ISDN > PBO.

**Table 16:** Time subgroup: league table of all-cause bleeding rates for 13-24 months.

FZHYJN+PPL										
0.53 (0.09, 3.20)	Carv									
0.49 (0.12, 2.00)	0.92 (0.19, 4.46)	FZHYJN								
0.28 (0.06, 1.29)	0.53 (0.19, 1.50)	0.58 (0.17, 2.02)	EVBL							
0.26 (0.06, 1.04)	0.49 (0.15, 1.55)	0.53 (0.17, 1.63)	0.91 (0.48, 1.73)	PPL						
0.23 (0.04, 1.38)	0.44 (0.10, 1.97)	0.48 (0.10, 2.23)	0.83 (0.27, 2.51)	0.91 (0.29, 2.83)	Nado					
0.18 (0.01, 4.52)	0.34 (0.02, 7.58)	0.37 (0.02, 8.13)	0.63 (0.03, 12.05)	0.69 (0.04, 13.11)	0.76 (0.04, 16.22)	Simv				
0.17 (0.03, 0.90)	0.31 (0.08, 1.31)	0.34 (0.08, 1.42)	0.59 (0.21, 1.63)	0.64 (0.23, 1.78)	0.71 (0.19, 2.72)	0.93 (0.05, 18.29)	EIS			
0.12 (0.01, 1.37)	0.24 (0.03, 1.97)	0.26 (0.03, 2.40)	0.44 (0.07, 2.83)	0.48 (0.07, 3.44)	0.53 (0.06, 4.64)	0.70 (0.02, 22.74)	0.75 (0.09, 6.23)	Nado+ISDM		
0.11 (0.02, 0.63)	0.21 (0.05, 0.93)	0.22 (0.05, 1.04)	0.38 (0.12, 1.21)	0.42 (0.14, 1.23)	0.46 (0.12, 1.80)	0.61 (0.03, 13.47)	0.65 (0.16, 2.67)	0.87 (0.10, 7.71)	ISDN	
0.10 (0.02, 0.45)	0.19 (0.05, 0.65)	0.21 (0.06, 0.68)	0.35 (0.17, 0.73)	0.39 (0.19, 0.78)	0.43 (0.14, 1.26)	0.56 (0.03, 9.71)	0.60 (0.26, 1.39)	0.80 (0.11, 5.87)	0.92 (0.28, 3.05)	PBO

##### 3.5.1.3 Over 24 months group

**Figure 18:**
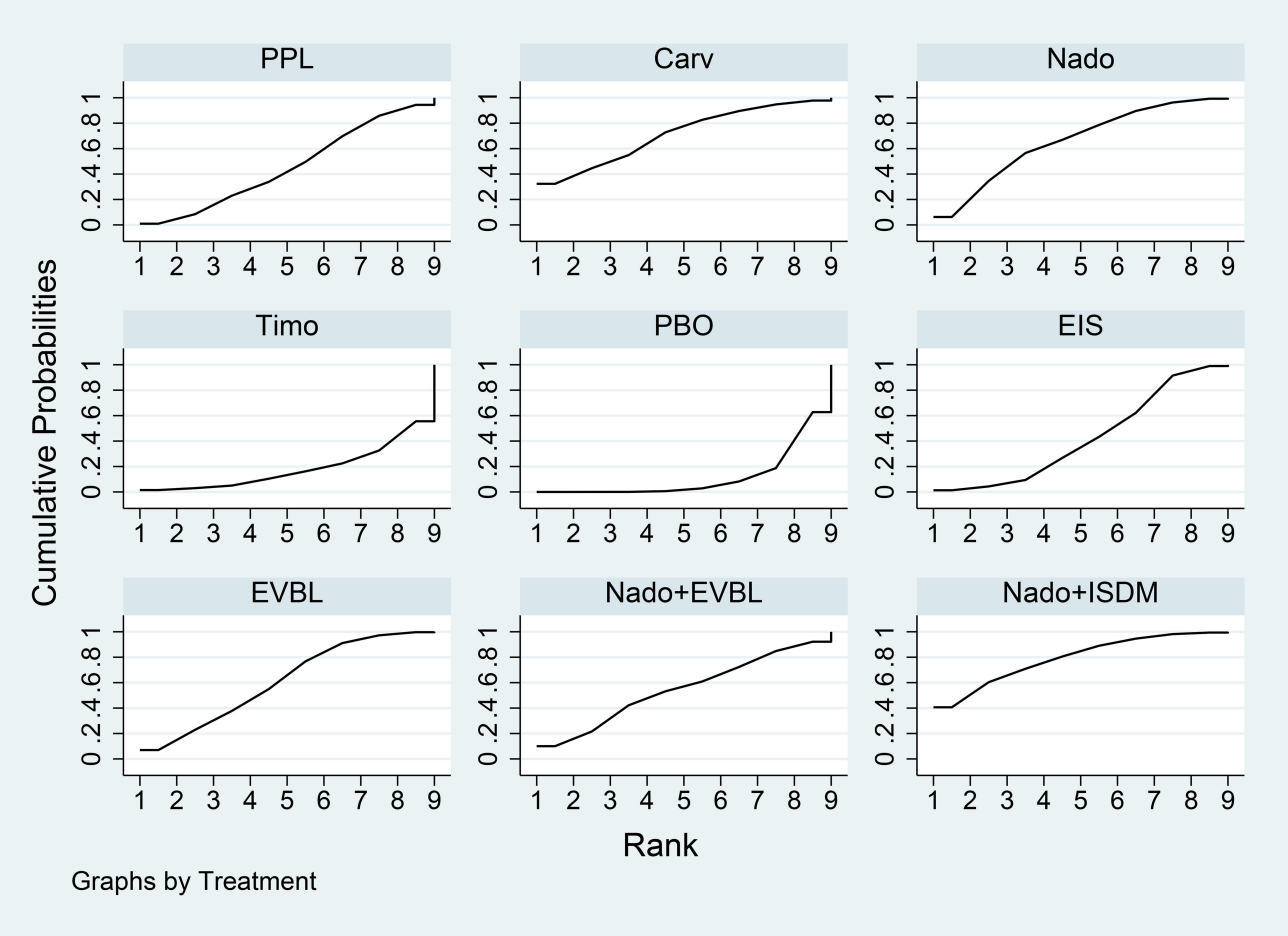
Time subgroup: all-cause bleeding rates for months over 24 SUCRA plot.

A total of 14 studies were conducted over a period exceeding two years, all of which were two-arm studies. These studies involved 1722 patients and evaluated 9 prophylactic interventions. The network meta-analysis results (**Table 17**) indicated that all interventions demonstrated statistically significant superiority over placebo. Compared with placebo, Nado+ISDM, Carv, and Nado were associated with significantly lower all-cause bleeding rates, with Nado+ISDM showing the lowest rate in the group exceeding two years. For NSBB outcomes, the ranking was Carv > Nado > PPL > Timo, with statistically significant differences observed. The cumulative ranking probability curve (**Figure 18**) demonstrated a larger area under the curve corresponds to a higher probability of benefit for the corresponding outcome. The ranking of interventions based on the probability of preventing bleeding beyond two years was as follows: Nado+ISDM > Carv > Nado > EVBL > Nado+EVBL > PPL > EIS > Timo > PBO.

**Table 17:** Time subgroup: league table of all-cause bleeding rates for months over 24.

Nado+ISDM								
0.64 (0.01, 43.39)	Carv							
0.59 (0.16, 2.18)	0.92 (0.02, 50.31)	Nado						
0.38 (0.01, 16.03)	0.60 (0.09, 4.17)	0.65 (0.02, 21.39)	EVBL					
0.39 (0.04, 3.61)	0.61 (0.01, 48.82)	0.66 (0.11, 4.00)	1.02 (0.02, 51.98)	Nado+EVBL				
0.27 (0.01, 12.46)	0.41 (0.08, 2.25)	0.45 (0.01, 16.76)	0.69 (0.27, 1.77)	0.68 (0.01, 38.86)	PPL			
0.20 (0.01, 5.28)	0.31 (0.02, 4.38)	0.34 (0.02, 6.80)	0.52 (0.09, 3.14)	0.51 (0.02, 17.02)	0.76 (0.10, 5.73)	EIS		
0.06 (0.00, 2.59)	0.09 (0.00, 3.86)	0.10 (0.00, 3.47)	0.16 (0.01, 3.75)	0.15 (0.00, 8.11)	0.22 (0.01, 6.23)	0.30 (0.02, 4.13)	Timo	
0.06 (0.00, 1.18)	0.09 (0.00, 1.73)	0.10 (0.01, 1.47)	0.15 (0.02, 1.40)	0.15 (0.01, 3.86)	0.22 (0.02, 2.45)	0.29 (0.08, 1.07)	0.98 (0.10, 9.67)	PBO

#### 3.5.2 Esophageal variceal bleeding rate

To evaluate whether the efficacy of pharmacological primary prophylaxis was consistent across patients with different stages of liver cirrhosis, we stratified the included RCTs based on the severity of cirrhosis—specifically Child-Pugh class A-B and class C—for the outcome of EVB rate. This subgroup analysis aimed to investigate the influence of varying degrees of liver cirrhosis on bleeding events.

##### 3.5.2.1 Child-Pugh class A–B

**Figure 19:**
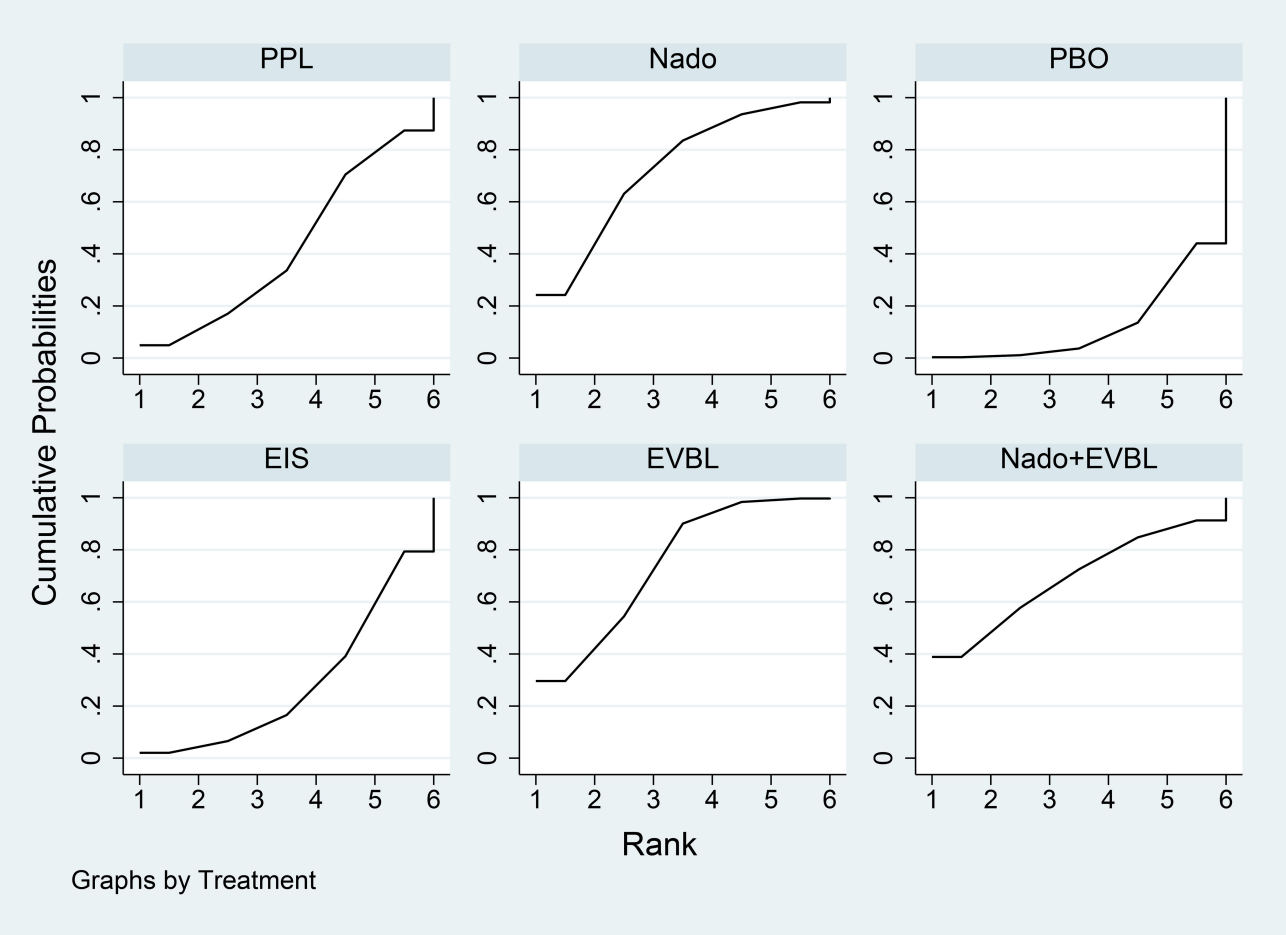
Liver function subgroup: league table of EVB rates for child-pugh class A-B SUCRA plot.

A total of 11 studies involving patients classified as Child-Pugh A-B were included, all of which were two-arm studies. These studies enrolled 980 patients and evaluated 6 prophylactic interventions. The network meta-analysis results (**Table 18**) demonstrated that all interventions were statistically significantly superior to placebo. Compared with placebo, EVBL, Nado, and Nado+EVBL were associated with significantly lower esophageal variceal bleeding (EVB) rates in Child-Pugh A-B patients, with EVBL showing the lowest EVB rate in this subgroup. For NSBB outcomes, the ranking was Nado > PPL, with statistically significant differences. The cumulative ranking probability curve (**Figure 19**) indicated a larger area under the curve corresponds to a higher probability of benefit for the corresponding outcome. The ranking of interventions based on the probability of reducing EVB in Child-Pugh A/B patients was as follows: EVBL > Nado > Nado+EVBL > PPL > EIS > PBO.

**Table 18:** Liver function subgroup: league table of EVB rates for Child-Pugh class A–B.

EVBL					
1.07 (0.22, 5.35)	Nado				
1.09 (0.11, 11.22)	1.02 (0.19, 5.49)	Nado+EVBL			
0.51 (0.16, 1.60)	0.48 (0.07, 3.04)	0.47 (0.04, 5.72)	PPL		
0.35 (0.09, 1.43)	0.33 (0.05, 2.13)	0.32 (0.03, 4.00)	0.69 (0.14, 3.42)	EIS	
0.25 (0.07, 0.96)	0.24 (0.04, 1.34)	0.23 (0.02, 2.61)	0.50 (0.11, 2.23)	0.72 (0.25, 2.04)	PBO

##### 3.5.2.2 Child-Pugh class C

A total of 10 studies involving patients classified as Child-Pugh C were included, comprising 1 single-arm study, 8 two-arm studies, and 1 three-arm study. These studies enrolled 235 patients and evaluated 6 prophylactic interventions. The network meta-analysis results (**Table 19**) indicated that all interventions demonstrated statistically significant superiority over Nado+EVBL. The inferior performance of nadolol compared to placebo may be attributed to the following reasons: (1) In patients with end-stage cirrhosis (e.g., Child-Pugh class C or refractory ascites), nadolol may reduce cardiac output and exacerbate renal hypoperfusion, thereby counteracting its potential benefit in bleeding prevention (113). (2) The efficacy of nadolol was dose-dependent. Previous studies have shown that a daily dose ≥60 mg significantly reduces bleeding risk (adjusted HR = 0.64), whereas doses ≤40 mg exhibit limited effectiveness (114). Notably, the RCTs included in this analysis employed low-dose timolol (40 mg/day), which may also contribute to its suboptimal overall performance.

**Table 19:** Liver function subgroup: league table of EVB rates for Child-Pugh class C.

EVBL					
0.88 (0.28, 2.80)	EIS				
0.77 (0.22, 2.64)	0.87 (0.22, 3.46)	PPL			
0.62 (0.15, 2.59)	0.71 (0.19, 2.59)	0.81 (0.19, 3.42)	PBO		
0.21 (0.02, 2.52)	0.24 (0.02, 3.68)	0.27 (0.02, 4.38)	0.33 (0.02, 5.91)	Nado	
0.11 (0.00, 2.74)	0.13 (0.00, 3.80)	0.14 (0.00, 4.49)	0.18 (0.01, 5.96)	0.53 (0.07, 4.01)	Nado+EVBL

**Figure 20:**
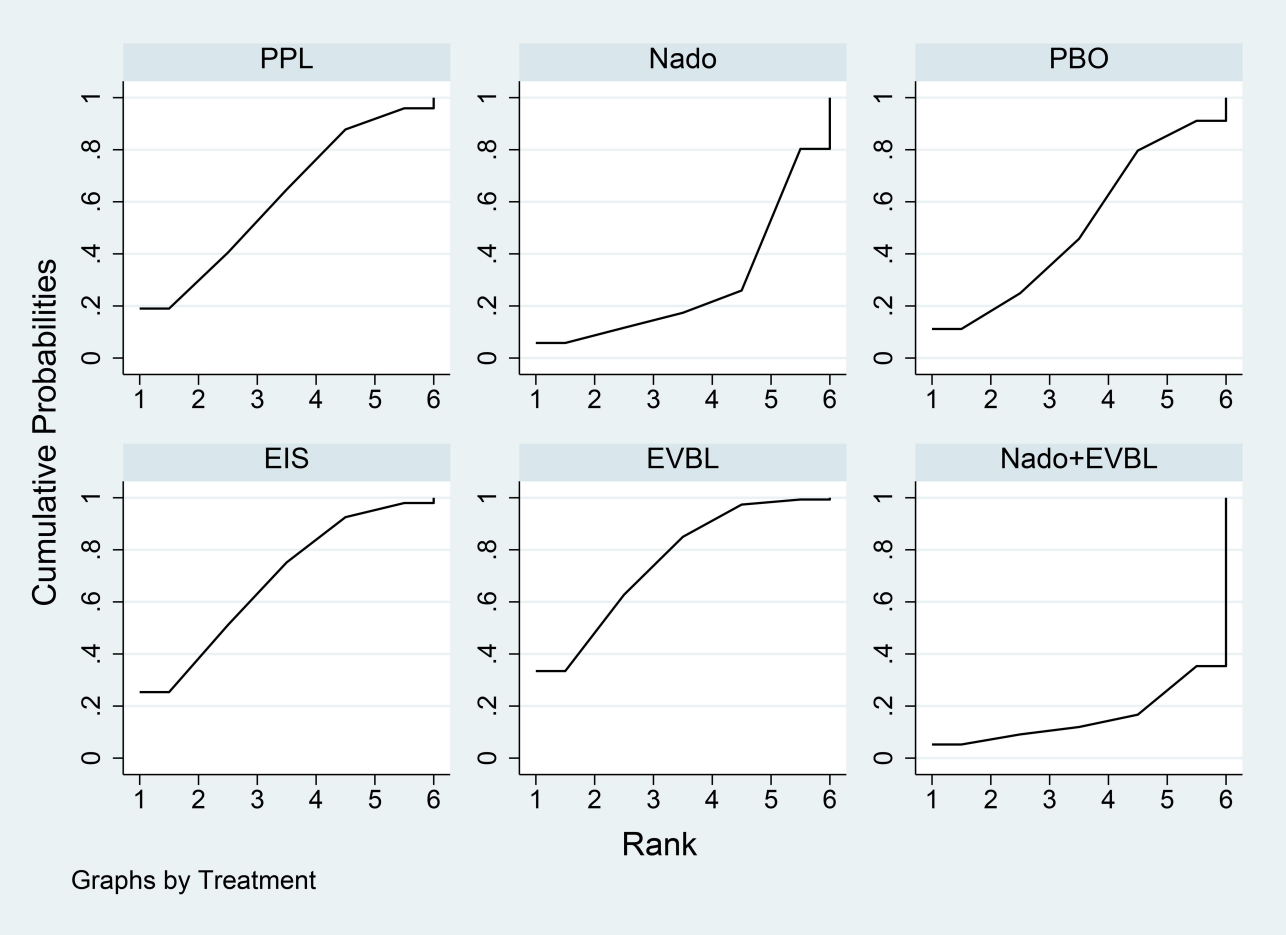
Liver function subgroup: league table of EVB rates for child-pugh class C SUCRA plot.

Compared with placebo, EVBL, EIS, and PPL were associated with significantly lower EVB rates in Child-Pugh C patients, with EVBL showing the lowest EVB rate in this subgroup. For NBSS outcomes, the ranking was PPL > Nado, with statistically significant differences. The cumulative ranking probability curve (**Figure 20**) demonstrated that a larger area under the curve corresponds to a higher probability of benefit for the corresponding outcome. The ranking of interventions based on the probability of reducing EVB in Child-Pugh C patients was as follows: EVBL > EIS > PPL > PBO > Nado > Nado+EVBL.

To evaluate the consistency of efficacy of study drugs for primary prophylaxis across patients with varying degrees of esophageal varices, we stratified the included RCTs based on variceal severity—specifically, mild-to-moderate and severe varices—for the outcome of EVB rate. This subgroup analysis aimed to investigate the influence of different variceal severity levels on bleeding events.

#### 3.5.3 Esophageal variceal bleeding rate

##### 3.5.3.1 Mild-to-moderate varices

**Figure 21:**
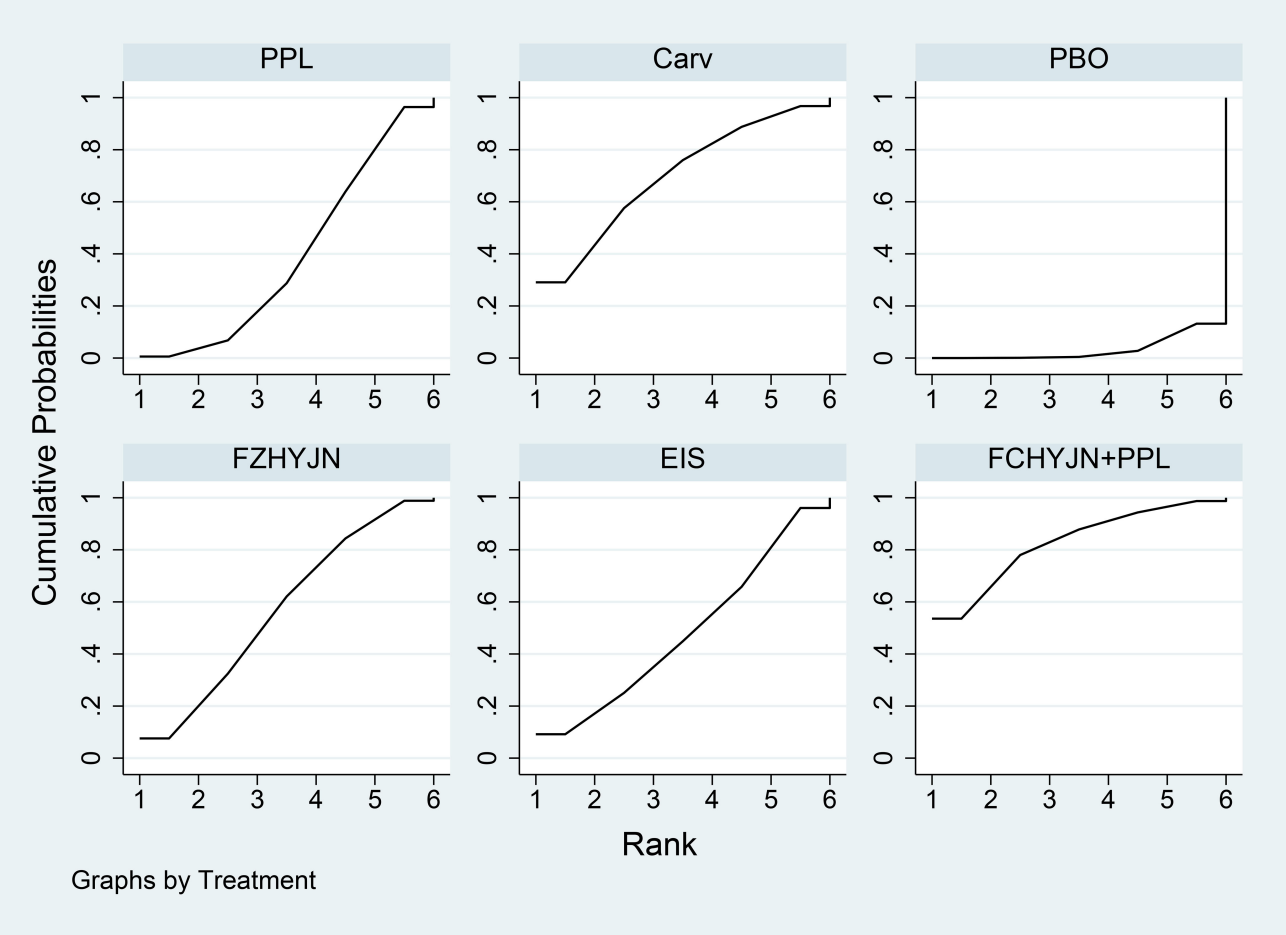
Variceal subgroup: EVB rates in patients with mild to moderate varices SUCRA plot.

A total of 7 studies involving patients with mild-to-moderate varices were included, comprising 6 two-arm studies and 1 three-arm study. These studies enrolled 506 patients and evaluated 6 prophylactic interventions. The network meta-analysis results (**Table 20**) indicated that all interventions were statistically significantly superior to placebo. Compared with placebo, FZHYJN+PPL, Carv, and FZHYJN were associated with significantly lower EVB rates in patients with mild-to-moderate varices, with FZHYJN+PPL showing the lowest EVB rate in this subgroup. For NBSS outcomes, the ranking was Carv > PPL, with statistically significant differences. The cumulative ranking probability curve (**Figure 21**) demonstrated that a larger area under the curve corresponds to a higher probability of benefit for the corresponding outcome. The ranking of interventions based on the probability of reducing EVB in patients with mild-to-moderate varices was as follows: FZHYJN+PPL > Carv > FZHYJN > EIS > PPL > PBO.

**Table 20:** Variceal subgroup: league table of EVB rates in patients with mild to moderate varices.

FZHYJN+PPL					
0.55 (0.02, 12.61)	Carv				
0.34 (0.03, 3.98)	0.62 (0.04, 8.84)	FZHYJN			
0.25 (0.01, 5.39)	0.46 (0.03, 8.41)	0.75 (0.07, 7.43)	EIS		
0.21 (0.02, 2.42)	0.38 (0.05, 2.70)	0.61 (0.10, 3.68)	0.82 (0.10, 6.96)	PPL	
0.06 (0.00, 0.81)	0.10 (0.01, 1.30)	0.16 (0.03, 0.87)	0.22 (0.04, 1.21)	0.27 (0.05, 1.37)	PBO

##### 3.5.3.2 Severe varices

**Figure 22:**
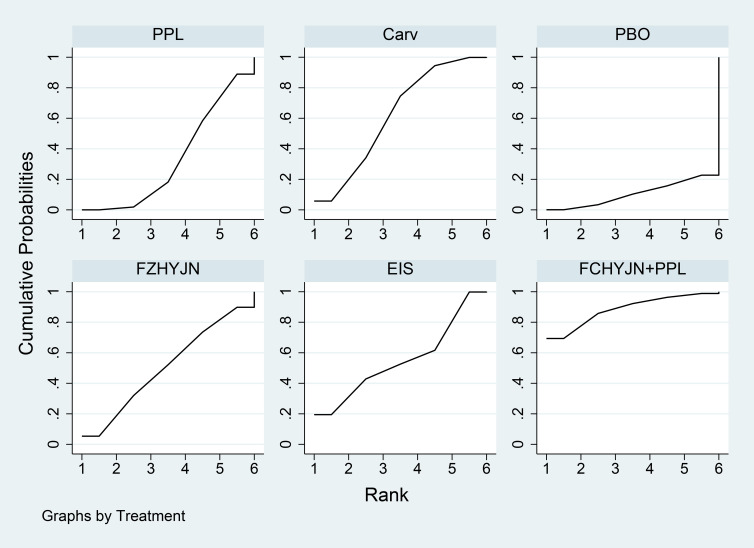
Variceal subgroup: EVB rates in patients with severe varices SUCRA plot.

A total of 6 studies involving patients with severe varices were included, consisting of 2 single-arm studies, 3 two-arm studies, and 1 three-arm study. These studies enrolled 207 patients and evaluated 6 prophylactic interventions. The network meta-analysis results (**Table 21**) demonstrated that all interventions were statistically significantly superior to placebo. Compared with placebo, FZHYJN+PPL, Carv, and EIS were associated with significantly lower EVB rates in patients with severe varices, with FZHYJN+PPL showing the lowest EVB rate in this subgroup. For NSBB outcomes, the ranking was Carv > PPL, with statistically significant differences. The cumulative ranking probability curve (**Figure 22**) indicated that a larger area under the curve corresponds to a higher probability of benefit for the corresponding outcome. The ranking of interventions based on the probability of reducing EVB in patients with severe varices was as follows: FZHYJN+PPL > Carv > EIS > FZHYJN > PPL > PBO.

**Table 21:** Variceal subgroup: league table of EVB rates in patients with severe varices.

FZHYJN+PPL					
0.43 (0.10, 1.84)	Carv				
0.40 (0.04, 4.30)	0.93 (0.14, 6.21)	EIS			
0.40 (0.09, 1.75)	0.92 (0.35, 2.44)	1.00 (0.12, 8.26)	FZHYJN		
0.33 (0.08, 1.39)	0.77 (0.62, 0.95)	0.83 (0.13, 5.51)	0.83 (0.32, 2.15)	PPL	
0.14 (0.01, 1.47)	0.33 (0.05, 2.11)	0.36 (0.17, 0.75)	0.36 (0.05, 2.84)	0.43 (0.07, 2.70)	PBO

### 3.6 Publication bias

**Figure 23:**
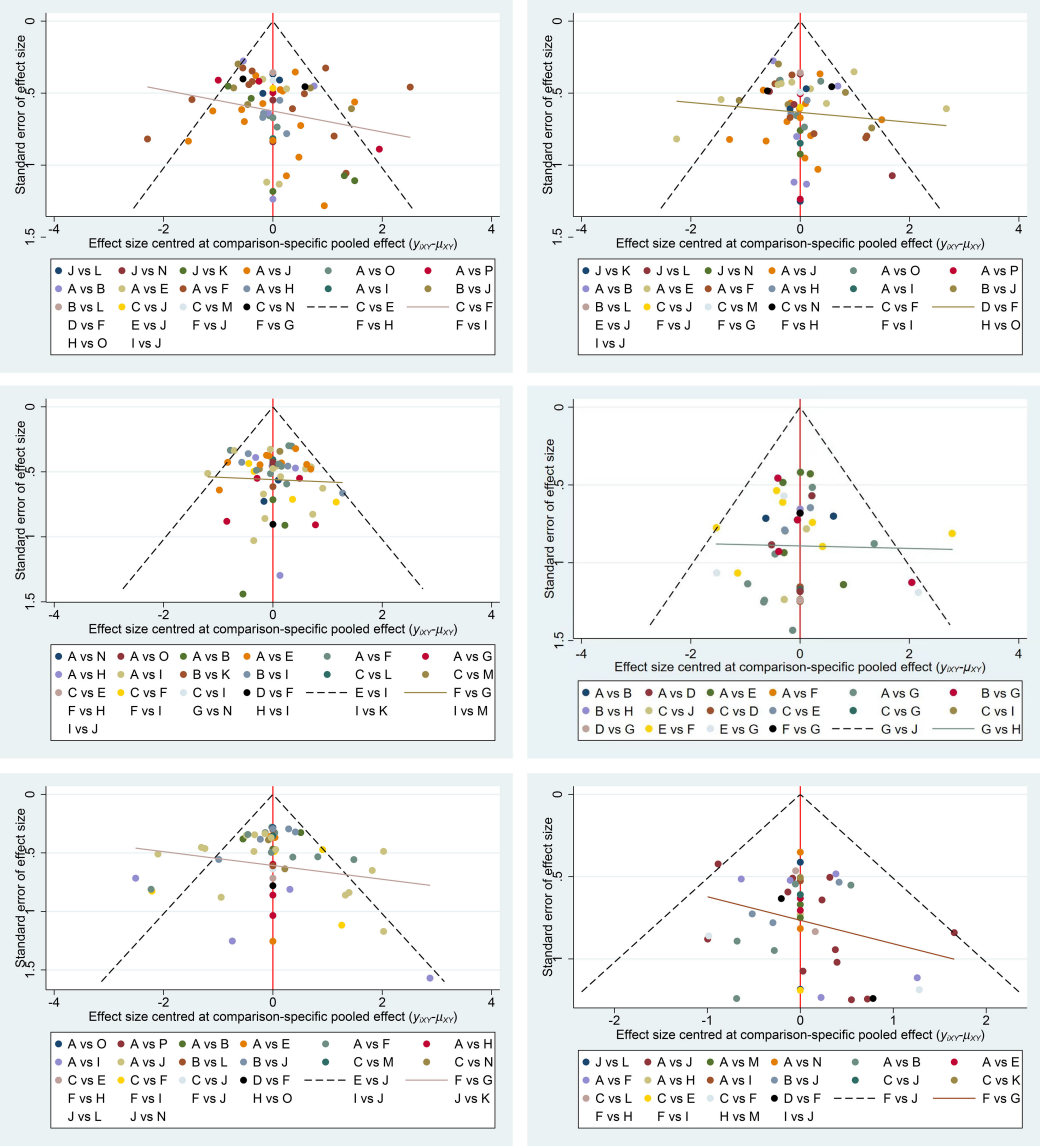
Risk of bias summary figure. A,PPL, B,Carv, C,Nado, D,Timo, E,ISDN, F,PBO, G,Simv, H,FZHYJN, I,EIS, J,EVBL, K,PPL+EVBL, L,Carv+EVBL, M,Nado+EVBL, N,Nado+ISDM, O,FZHYJN+PPL, P, Mido+PPL.

Funnel plot and Egger’s regression test were performed to assess publication bias for the primary outcomes. The data points were distributed approximately symmetrically about the axis, with no significant asymmetry detected, suggesting a low likelihood of publication bias (**Figure 23**). Egger's test results are presented in **Table 22**. The P-values for all indicators are greater than 0.05, indicating no significant publication bias.

**Table 22:** Egger’s test P-values by index.

Index	P-value of Egger’s test
All-Cause Bleeding Rates	0.111
Esophageal Variceal Bleeding (EVB) Rate	0.076
All-Cause Mortality Rate	0.508
Bleeding-Related Mortality Rate	0.903
Incidence of General Adverse Events Rate	0.487
Incidence of Other Decompensation Events Rate	0.132

## 4 Discussion

The meta-analysis revealed that regarding bleeding suppression efficacy, several combination regimens demonstrated notably lower bleeding rates for both esophageal and gastric varices. Specifically, the Mido+PPL, Carv+EVBL, and FZHYJN+PPL schemes were associated with the most favorable outcomes. These combination strategies demonstrated superior efficacy in preventing variceal hemorrhage compared to the conventional monotherapies of either EVBL or NSBB alone, which are currently recommended as standard first-line interventions by major guidelines (7–12, 115). Experimental and clinical studies have demonstrated that FZHYJN significantly reduces portal venous pressure by improving intrahepatic microcirculation and inhibiting hepatic stellate cell activation (116–118). To our knowledge, this is the first robust evidence indicating its efficacy in primary prevention of EVB, which is comparable to that of NSBB or EVBL. Midodrine, an α-1 adrenergic agonist, enhances renal perfusion and systemic hemodynamics by counteracting splanchnic vasodilation commonly associated with advanced cirrhosis (119). Emerging evidence indicates that adjunctive use of midodrine in patients with cirrhosis and refractory ascites facilitates uptitration to the maximum tolerated dose (MTD) of NSBB like propranolol, thereby augmenting the reduction of portal pressure and mitigating the risk of variceal hemorrhage. Furthermore, combination therapy incorporating midodrine demonstrates superior efficacy in primary prevention of EVB, significantly lowering the incidence of bleeding events compared to NSBB monotherapy (120, 121). Regarding the endpoint of all-cause bleeding, current guidelines and literature provide no definitive recommendation on the optimal duration for primary prophylaxis, nor do they elucidate whether the effectiveness of various preventive strategies is time-dependent. To address this gap, our study is the first to categorize the included RCTs into three distinct temporal subgroups for analysis: 1–12 months, 13–24 months, and beyond 24 months, aiming to evaluate the effects of different prophylaxis durations on bleeding prevention. The analysis of temporal trends revealed distinct profiles in all-cause bleeding rates across various prophylactic regimens: Within 1–12 months, EIS, Mido+PPL, and PPL+EVBL demonstrated notably low bleeding rates. During the 13–24 months, FZHYJN+PPL, Carv, and FZHYJN were associated with superior bleeding prevention. Beyond 24 months, the Nado+ISDM, Carv, and Nado exhibited the most sustained efficacy in maintaining low bleeding rates. Regarding the included RCTs, it was noted that the dosage of nadolol administered was relatively low, potentially falling within a subtherapeutic range. In contrast, the combination therapy involving ISDN and NSBB demonstrated a significant long-term benefit in mitigating the progression of portal hypertension. This synergistic effect is primarily mediated through a sustained reduction in Hepatic Venous Pressure Gradient (HVPG), which consequently diminishes wall tension in vasculature and reduces persistent hemodynamic stress on variceal walls, thereby effectively lowering the risk of rupture (122). Furthermore, the efficacy of this combined regimen, particularly the ISDN and Nado strategy, appears to exhibit a time-dependent cumulative therapeutic advantage, suggesting that extended duration of prophylaxis is associated with superior clinical outcomes in preventing variceal bleeding. The league table analysis from the network meta-analysis revealed time-dependent efficacy profiles for primary prevention strategies regarding all-cause bleeding:Within 1–12 months, the combination of Mido+PPL was associated with the lowest incidence of all-cause bleeding. During the 13–24 months, the regimen FZHYJN+PPL demonstrated superior efficacy in minimizing bleeding events. Beyond 24 months of continuous prophylaxis, the strategy of ISDN+Nado showed the most sustained protective effect against all-cause bleeding. These findings highlight the importance of considering temporal dynamics when selecting long-term prophylactic strategies for variceal bleeding in cirrhotic patients.

Current literatures lack comprehensive stratification based on the severity of liver dysfunction in evaluating primary prophylactic regimens for esophageal variceal bleeding, particularly regarding the comparative efficacy of combination therapies across different patient subgroups. To address this gap, our study is the first to categorize patients according to Child-Pugh classification into two distinct cohorts: Child-Pugh A-B and Child-Pugh C groups, aiming to elucidate the differential effectiveness of various preventive strategies, including novel combination approaches in these populations. According to the network meta-analysis, distinct profiles of prophylactic efficacy against EVB were observed between patients with different liver function reserves classified by Child-Pugh grade:In patients with Child-Pugh grade A-B, EVBL, Nado monotherapy, and the combination of Nado+EVBL were associated with relatively lower EVB rates. Among these interventions, EVBL demonstrated the lowest incidence of EVB. In patients with Child-Pugh grade C, the strategies of EVBL, EIS, and PPL monotherapy were linked to lower EVB rates. Similarly, EVBL emerged as the most effective measure in preventing EVB within this high-risk subgroup. In the subgroup analyses stratified by cirrhosis severity (e.g., Child-Pugh classification), the regimens Mido+PPL, Carv+EVBL, and FZHYJN+PPL were not included in the comparative outcomes. This omission primarily stems from the fact that the original RCTs investigating these specific interventions did not pre-specify or stratify their study populations based on liver cirrhosis severity grades (15, 50, 56). Consequently, the necessary data to evaluate their efficacy across different Child-Pugh categories (e.g., Class A-B vs. Class C) is lacking, preventing meaningful subgroup comparisons or meta-analytic conclusions for these regimens in relation to baseline liver function reserve.

The study is the first to categorize patients into distinct subgroups—namely, a mild-to-moderate variceal group and a severe variceal group—based on endoscopic appearance and hemorrhage risk features. This approach allows for a detailed investigation into the differential effectiveness of primary prevention strategies across varying levels of variceal burden. According to the network meta-analysis, distinct efficacy profiles of prophylactic regimens were observed between patients with mild-to-moderate and severe esophageal varices: In patients with mild-to-moderate varices, the regimens of FZHYJN+PPL, Carv, and FZHYJN were associated with relatively lower rates of EVB. Among these, the FZHYJN+PPL combination demonstrated the lowest incidence of EVB. In patients with severe varices, the strategies involving FZHYJN+PPL, Carv, and EIS were linked to superior bleeding prevention. Similarly, the FZHYJN+PPL regimen again emerged as the most effective intervention with the lowest EVB rate in this high-risk subgroup. These subgroup findings suggest that the FZHYJN+PPL exhibits broad-spectrum applicability across various stages of variceal severity, consistently outperforming other monotherapies and even some combined approaches. Given its robust efficacy profile, this regimen—though not yet incorporated into major guidelines—may represent a promising novel first-line preventive strategy for patients with cirrhosis at risk of initial variceal hemorrhage.

Regarding the efficacy of NSBB, the relative performance of various agents differed across distinct clinical endpoints based on our analysis: For all-cause bleeding, the efficacy hierarchy was observed as follows: Carv > PPL> Nado > Timo. Concerning EVBL, the order of effectiveness was: Carv > PPL > Timo > Nado. In terms of all-cause mortality, the outcomes favored: PPL > Carv > Timo > Nado. Specifically for bleeding-related mortality, the results indicated: Carv > PPL > Nado. A consistent pattern emerged from these findings: both carvedilol and propranolol consistently demonstrated superior efficacy across multiple endpoints compared to nadolol and timolol (123). This observed superiority aligns with and reinforced the current therapeutic recommendations outlined in European clinical guidelines for the management of portal hypertension and variceal bleeding (115, 124).

Regarding overall safety profiles, the regimens of Mido+PPL, Nado, and Carv were associated with a relatively lower incidence of general adverse events, among which the Mido+PPL combination demonstrated the most favorable tolerability. In terms of the risk of inducing other decompensation events in cirrhosis, ISDM, Mido+PPL, and Carv were linked to a lower frequency, with ISDM monotherapy showing the lowest incidence. It is noteworthy that these findings diverge from conclusions reported in several previous studies, highlighting the need for further investigation into patient selection, dosing strategies, and underlying mechanistic interactions that may explain these differences in safety outcomes (10, 125). Regarding safety profiles, the FZHYJN+PPL regimen was associated with a higher incidence of decompensation events, indicating that the safety of traditional Chinese medicine formulation FZHYJN requires further validation in larger and more rigorously designed studies (126).

Compared with previously published network meta-analyses on the same topic, the present study incorporates several novel combination strategies for the primary prophylaxis of esophageal variceal bleeding in cirrhosis (e.g. Carv+EVBL, Mido+PPL and FZHYJN+PPL), thereby enabling a broader and more comprehensive evaluation of the relative efficacy of various prophylactic interventions (5, 14). This expanded analytical approach offers enhanced evidence to support clinical decision-making and provides a wider range of therapeutic options for personalized patient management.

This study has several limitations that should be considered when interpreting the findings. Firstly, the analysis primarily evaluated the efficacy of various interventions on seven outcome measures, including all-cause bleeding, esophageal variceal bleeding, and all-cause mortality. The relative ranking of interventions based on these outcomes may not be consistent with their performance on other clinical endpoints not examined in this study. Secondly, due to insufficient descriptions of randomization procedures, blinding, and allocation concealment in some included trials, the potential for selection, performance, or detection bias cannot be ruled out. Thirdly, evidence on several combination prophylaxis strategies—such as Carv+EVBL, Mido+PPL, and FZHYJN+PPL—remain limited, as these regimens are investigated in a small number of trials with relatively limited patient populations. Furthermore, subgroup analyses are constrained by insufficient number of studies and small sample sizes, which may reduce the reliability and precision of subgroup-specific conclusions. Finally, the number of preventive strategies available for direct comparison within each subgroup is limited, thereby restricting the ability to comprehensively evaluate all regimens across different patient populations. Future studies should prioritize larger randomized trials with explicit methodology reporting, incorporate a broader range of outcome measures, and strive to include more diverse treatment regimens in subgroup analyses to enhance the generalizability and clinical applicability of the findings.

## 5 Conclusion

1. Based on analyses of low-certainty evidence, combined strategies such as Carv+EVBL, Mido+PPL, and FZHYJN+PPL demonstrated significant advantages over monotherapies (including NSBB or EVBL alone) in reducing all-cause bleeding, variceal hemorrhage, and all-cause mortality. These findings suggest that combination regimens, through multi-target synergistic effects, represent preferred strategies for high-risk patients (e.g., Child-Pugh class C), while EVBL monotherapy remains a viable alternative for those with contraindications to pharmacological therapy.
2. This study pioneered temporal subgroup analysis (1-12/13-24/>24 months), revealing that the superiority of prophylactic interventions is duration-dependent. Based on low-certainty evidence, Mido+PPL was most effective for short-term prevention (≤1 year), FZHYJN+PPL for medium-term (1–2 years), and ISDN+Nado for long-term maintenance (>2 years).
3. Subgroup analyses indicate that optimal prophylaxis requires customization based on liver disease severity and variceal grade. For Child-Pugh A-B patients, EVBL, Nado, or their combination showed efficacy, while EVBL, EIS, or PPL were preferable for Child-Pugh C patients. Regarding variceal severity, FZHYJN+PPL, Carv, or FZHYJN alone were effective for mild-moderate cases, whereas FZHYJN+PPL, Carv, or EIS were suitable for severe varices.
4. Analyses based on low-certainty evidence indicate that Carv and PPL are superior to Nado and Timo for primary prophylaxis of esophageal variceal bleeding.
5. Safety is paramount in intervention selection. Low-certainty evidence suggests Mido+PPL and Carv monotherapy have fewer general adverse events, while ISMN may delay decompensation events like ascites and SBP. Notably, FZHYJN+PPL was associated with higher decompensation rates, necessitating safety monitoring for long-term TCM use and further pharmacological validation.
