# Supplementary material for: Primary prevention in liver cirrhosis patients with esophageal varicesa: a systematic review and network meta-analysis
Source: Front Gastroenterol (Lausanne). 2026 Apr 16;5:1754027. doi: 10.3389/fgstr.2026.1754027 (PMC13128573; doi:10.3389/fgstr.2026.1754027)
Supplement: Supplementary file 1 [file Table1.docx]

**"Literature Search Strategy**

**pubmed**

**Search: ((((((Liver Cirrhosis) OR (Cirrhosis, Liver)) OR (Fibrosis, Liver)) OR (Hepatic Cirrhosis)) OR (Liver Fibrosis)) OR (Cirrhosis, Hepatic)) AND (esophageal varices)**

**result：****7705**

**Cochrane**

**#1**  **Liver Cirrhosis or Cirrhosis, Liver or Fibrosis, Liver or Hepatic Cirrhosis or Liver Fibrosis or Cirrhosis, Hepatic**

**#2 esophageal varices**

**#3 #1AND#2**

**result：1007**

**Web of science**

**#1 "Liver Cirrhosi*"OR "Cirrhosi*, Liver" OR "Fibrosi*, Liver" OR "Hepatic Cirrhosi*" OR "Liver Fibrosi*"OR "Cirrhosi*, Hepatic"**

**#2 (esophageal varice*)**

**#3 #1AND#2**

**result：8990**

**Embase**

**#5 #3 AND #4 6,427**

**#4 #1 OR #2 264,447**

**#3 'esophageal varices':ti,ab,kw 12,544**

**#2 'liver cirrhosis':ti,ab,kw OR 'cirrhosis, liver':ti,ab,kw OR 'fibrosis, liver':ti,ab,kw OR 'hepatic cirrhosis':ti,ab,kw OR 'liver fibrosis':ti,ab,kw OR 'cirrhosis, hepatic':ti,ab,kw 106,034**

**#1 'liver cirrhosis'/exp 223,069**

**result：6427**
